# Genetic differentiation and local adaptation of the Japanese honeybee, *Apis cerana japonica*

**DOI:** 10.1002/ece3.10573

**Published:** 2023-09-29

**Authors:** Takeshi Wakamiya, Takahiro Kamioka, Yuu Ishii, Jun‐ichi Takahashi, Taro Maeda, Masakado Kawata

**Affiliations:** ^1^ Graduate School of Life Sciences Tohoku University Sendai Japan; ^2^ Department of Biological Sciences Tokyo Metropolitan University Hachioji Japan; ^3^ Faculty of Life Sciences Kyoto Sangyo University Kyoto Japan; ^4^ Institute for Agro‐Environmental Sciences (NIAES) NARO Tsukuba Japan

**Keywords:** environmental adaptation, local adaptation, population branch statistics, population genetic structure, whole genome analysis

## Abstract

We examine the population genetic structure and divergence among the regional populations of the Japanese honeybee, *Apis cerana japonica*, by re‐sequencing the genomes of 105 individuals from the three main Japanese islands with diverse climates. The genetic structure results indicated that these individuals are distinct from the mainland Chinese *A. cerana* samples. Furthermore, population structure analyses have identified three genetically distinct geographic regions in Japan: Northern (Tohoku‐Kanto‐Chubu districts), Central (Chugoku district), and Southern (Kyushu district). In some districts, “possible non‐native” individuals, likely introduced from other regions in recent years, were discovered. Then, genome‐wide scans were conducted to detect candidate genes for adaptation by two different approaches. We performed a population branch statistics (PBS) analysis to identify candidate genes for population‐specific divergence. A latent factor mixed model (LFMM) was used to identify genes associated with climatic variables along a geographic gradient. The PBS_max_ analysis identified 25 candidate genes for population‐specific divergence whereas the LFMM analysis identified 73 candidate genes for adaptation to climatic variables along a geographic gradient. However, no common genes were identified by both methods.

## Graphical abstract

In this study, genetic structure and local adaptation of the Japanese honeybee, *Apis cerana japonica* were examined by re‐sequencing the genomes of 105 individuals from the three main islands of Japan. We identified three genetically distinct geographic regions in Japan: Northern (Tohoku‐Kanto‐Chubu districts), Central (Chugoku district), and Southern (Kyushu district). In some districts, “possible non‐native” individuals who were likely introduced from other regions in recent years were discovered. Then, genome‐wide scans were conducted to detect candidate genes for adaptation by two different approaches.

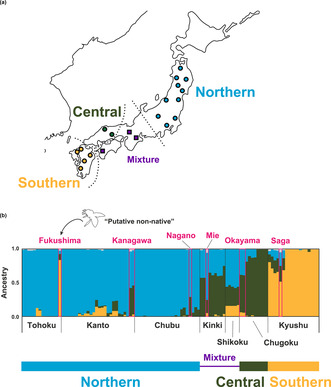

## 1 INTRODUCTION

An important question in evolution and conservation biology is what factors cause genetic differentiation among regions with a continuous distribution. Local adaptation can be indicated by genetic differentiation along environmental gradients (Savolainen et al., 2013). The ability to shift the distribution of populations responding to global climate change might depend on the local environmental factor the population is adapted to (Davis & Shaw, 2001). If populations are adapted to environmental variables along geographic gradients, individuals could shift their range along continuous gradients facing global climatic changes such as increasing temperatures. In contrast, local adaptation to historical conditions (e.g., geographic features) or non‐climatic environmental factors (e.g., soil conditions, predators, competitors, etc.) may increase vulnerability to climate change because it prevents organisms from shifting their habitats (Anderson & Wadgymar, 2020). Consequently, it is essential to determine the factors that cause local adaptation in each population.

Insect populations in Japan are a suitable subject for research on local adaptation due to the diverse climates and flora of the Japanese islands (Echenique‐Diaz & Yokoyama, 2009), which could drive insect population divergence among regions and local adaptation. For instance, a genome‐wide phylogeographic analysis of the Genji firefly (*Luciola cruciate*) identified three phylogenetic groups (East Honshu, West Honshu, and Kyushu; Kato et al., 2020); further, the growth rate of the rhinoceros beetle (*Trypoxylus dichotomus*) is strongly correlated with latitude (Kojima et al., 2020). Eastern honeybee (*Apis cerana*) is the only species of the genus *Apis* that is native to Japan. The Japanese honeybee (*A. c. japonica*) is found on the three main islands of Japan (i.e., Honshu, Kyushu, and Shikoku), as well as certain remote islands. Due to its cold tolerance, pollination ability, and defensive behavior against hornets, this subspecies has garnered considerable interest as a unique bioresource (Sasaki, 1999). The significance of *A. c. japonica* as pollinators in local ecosystems is becoming more widely recognized (Fujiwara & Washitani, 2016; Taki et al., 2010; Tatsuno & Osawa, 2016). Meanwhile, the population of this subspecies is reportedly declining (Theisen‐Jones & Bienefeld, 2016), highlighting the need for ongoing conservation efforts.

Although *A. c. japonica* is widely distributed throughout the various biomes of the Japanese islands, the population genetic structure and local adaptation of this subspecies have not been fully investigated. Previous research on the genetic structure of *A. c. japonica* indicated only weak population genetic differentiation using the mitochondrial DNA D‐loop region (Takahashi et al., 2007) and six microsatellite loci (Nagamitsu et al., 2016). Fujiwara et al. (2015) discovered no significant difference in body size among *A. c. japonica* populations, except for the Amami‐Oshima Island population. Nevertheless, these previous studies employed a limited numbers of genetic or morphometric markers, so large‐scale genomic data are required to evaluate the genetic divergence and local adaptation of *A. c. japonica*.

After publication of the first report of the genome sequence of *A. mellifera* (HGSC, 2006), the whole genomes of a large number of individuals were re‐sequenced, which led to rapid progress in studies of the genetic population structure and detection of genes under natural selection (Dogantzis & Zayed, 2019; Yunusbaev et al., 2019). Global‐scale genome analyses revealed the evolutionary history and local adaptation of different lineages of *A. mellifera* (Han et al., 2012; Wallberg et al., 2014; Whitfield et al., 2006). For instance, candidate genes for adaptation to high altitude (Wallberg et al., 2017), temperature (Chen et al., 2016), precipitation, longitude and latitude (Henriques et al., 2018), and social parasitism (Wallberg et al., 2016) have been detected.

While there have been numerous genomic studies of *A. mellifera*, those of the Asian honeybee species are relatively limited. The whole genome sequences of *A. cerana* and *A. dorsata* have been reported (e.g., Oppenheim et al., 2019; Park et al., 2015), and genome‐wide analyses of genes associated with local environments have been conducted (Chen et al., 2018; Ji et al., 2020; Montero‐Mendieta et al., 2019; Shi et al., 2020). Honeybee species in Asia are notable for their relatively mild domestication bias and wide range distribution as wild species. In *A. cerana*, the genomes of 180 individuals from 18 Chinese populations were sequenced (Chen et al., 2018); then, Montero‐Mendieta et al. (2019) identified candidate genes for adaptation to high altitudes. In addition, functional analyses of these several candidate genes, including *AcVIAAT* and the Leucokinin receptor (Ji et al., 2020; Shi et al., 2020), have been carried out. Most of these studies, however, have focused on the Chinese populations of *A. cerana* but have not examined any other subspecies. Yokoi et al. (2018) sequenced the *A. c. japonica* genome and described several distinct characteristics of this subspecies' genome compared to those of *A. cerana* (e.g., smaller number of mariner‐like transposable elements). *A. c. japonica* possesses numerous phenotypic (e.g., mildness, low honey production, pests–pathogens resistance, and frequent absconding) and genomic characteristics. Consequently, high‐resolution genomic analyses of this subspecies could reveal a distinct population genetic structure and adaptive evolution relative to other *Apis* species.

In this study, we investigate the population genetic structure and local adaptation of the regional populations of *A. c. japonica* by re‐sequencing the new genomes of 105 individuals from the three main islands of Japan. Three genetically diverged geographic regions within Japan were identified using genome‐wide single‐nucleotide polymorphisms (SNPs) of the *A. c. japonica* samples. Then, candidate genes for adaptation were detected using two different approaches. Population branch statistics (PBS) was used to identify candidate genes for population‐specific divergence (locally adapted genes), and a latent factor mixed model (LFMM) was employed to identify genes associated with climatic and environmental factors (temperature, snowfall, precipitation, and sunlight). Based on these results, we discuss the evolution of genetic divergence and local adaptation of this subspecies.

## 2 MATERIALS AND METHODS

### 2.1 Samples

**FIGURE 1:**
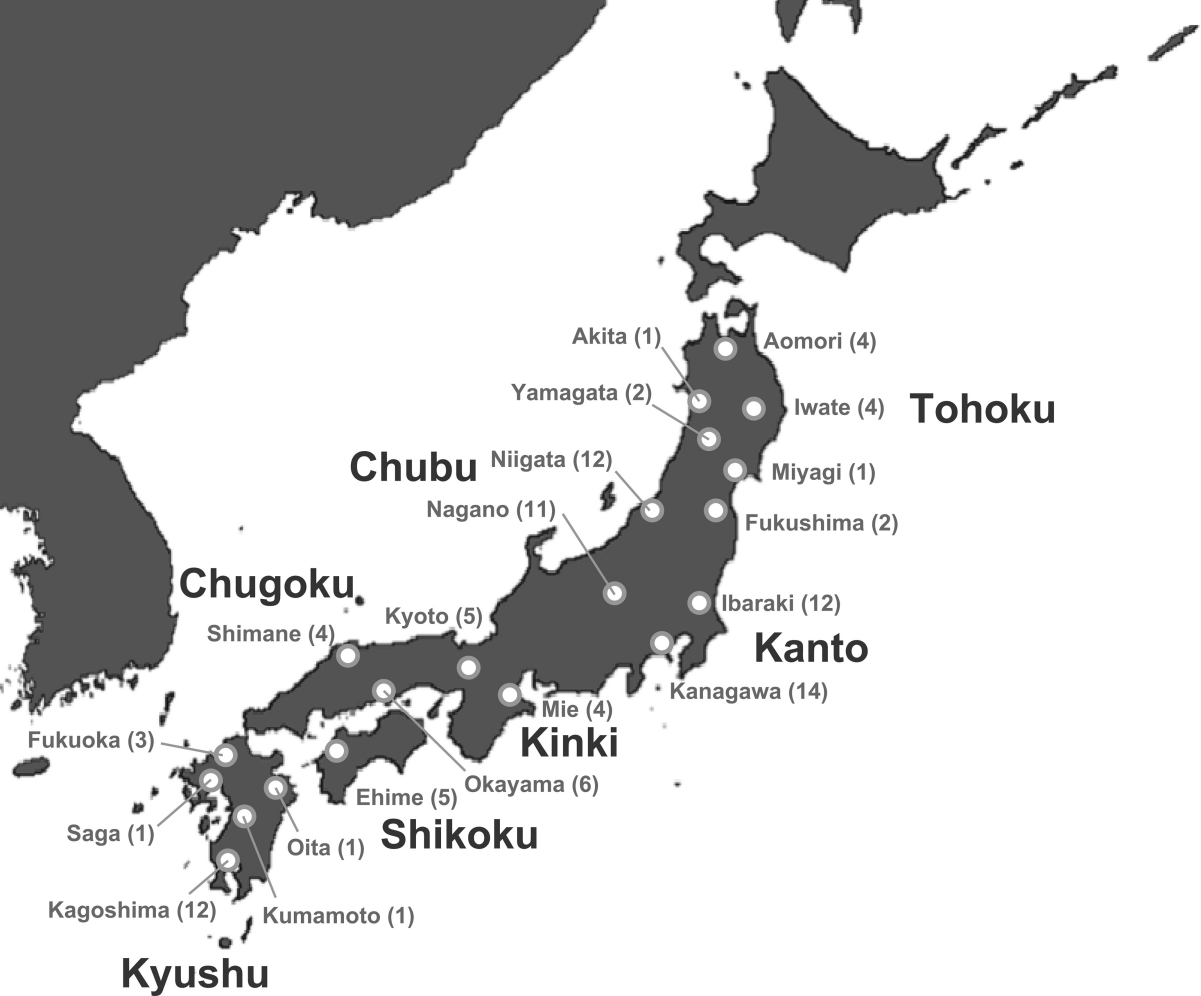
Map of the sampling locations. The numbers in parenthesis indicate the number of samples.

In total, 105 *A. c. japonica* worker individuals were collected from various locations on the Japanese islands with native *A. c. japonica* populations for genome sequencing (Figure 1; Table S1). Of these 105 individuals, 87 were provided by the National Agriculture and Food Research Organization (NARO; Tsukuba, Japan), and 18 were obtained from research collaborators. The Japanese samples were collected mainly from beekeepers. Japanese honeybee beekeeping is conducted by wild bees entering artificial hives. Therefore, the individuals collected from the hive are originated from wild individuals living in the vicinity. However, colonies are sometimes transferred from other locations. In this case, we confirmed the presence of “introduced individuals” by interviews with beekeepers.

In addition to Japanese samples, the genome sequence data of eight individuals of Chinese *A. cerana* (part of QY and YL populations, located in North East and North Central China, respectively; Chen et al., 2018) were downloaded from the National Center for Biotechnology Information Sequence Read Archive database to serve as reference population (Table S2). Five individuals from the Korean Peninsula (Ji et al., 2020) were also used to estimate the population genetic structure. The bioinformatics methods used in this study are summarized in Texts S1 and S2 in Data S1.

### 2.2 DNA extraction and next‐generation sequencing (NGS)

Genomic DNA was extracted from thoracic muscle tissue using the DNeasy Blood and Tissue Kit (QIAGEN). The extracted genomic DNA was processed using the TruSeq Nano DNA Low Throughput Library Prep Kit (Illumina, Inc.). The resulting libraries were outsourced to Macrogen Japan for sequencing, which was conducted using a HiSeq X Ten Sequencing System (Illumina Inc.; 150‐bp paired ends). A minimum of 6 Gbp were obtained per individual, which corresponds to approximately 29× coverage in terms of genome size for this subspecies (211 Mbp; Yokoi et al., 2018). The original NGS reads were registered with the DNA Data Bank of Japan database (under BioProject PRJDB14080, DRR397616‐DRR397720).

### 2.3 Quality control, variant calling, and SNP filtering

Raw NGS reads were quality‐controlled using the FastQC quality control tool (Andrews, 2010), the Fastx‐toolkit (http://hannonlab.cshl.edu/fastx_toolkit/) and a custom Perl script. Specifically, reads were excluded if 50% or more of the bases were graded “low quality” (i.e., Q score ≤ 30). Only resultant reads with both pairs maintained were mapped to the *A. c. japonica* reference genome sequence (Yokoi et al., 2018) using the BWA‐MEM algorithm (Li & Durbin, 2009). Resultant bam files were processed using SAMtools1.8 and BCFtools1.8 (Li et al., 2009), and variant calling was performed for each individual. For variant calling, the “samtools rmdup” command was used to remove PCR duplicates and the initial filtering was performed using the “vcfutils.pl varFilter ‐d 5 ‐D 100” command. Next, all variants were merged into a single vcf file using vcftools 0.1.16 (Danecek et al., 2011), which resulted in two datasets: the Japanese individual data with and without the Chinese reference population. Finally, after removing the insertion/deletion variants, the datasets were filtered with a minor allele frequency of 0.05.

### 2.4 Genetic structure and genetic diversity

The genetic structure was evaluated using ADMIXTURE software (Alexander et al., 2009) and principal components analysis (PCA) with the PLINK1.90 toolset (Purcell et al., 2007). Genetic structures were estimated using Japanese and Chinese samples first, and additionally, using Japanese, Chinese, and Korean samples. For genetic structure analyses, a linkage disequilibrium removal step was applied to the datasets using PLINK1.90 (‐‐indep‐pairwise 50 10 0.1; Purcell et al., 2007). At *K* (number of clusters) = 1–6, an ADMIXTURE analysis was conducted, and the cross‐validation (CV) error rate was calculated. PCA was performed on the Japanese individuals' dataset. Based on the outcomes of both population genetic structure analyses, individuals with ≥28% aberrant genetic composition compared to other members of the same district (“putative non‐native” individuals) were excluded from subsequent candidate gene analyses. In addition, vcftools 0.1.16 (Danecek et al., 2011) was used to calculate the basic genetic statistics (number of polymorphic sites, average nucleotide diversity (PI/10 kb), and mean *F*_ST_).

### 2.5 Candidate genes associated with local adaptation and environmental factors

PBS was used to examine the genomes of the three genetically divergent populations (the three genetically distinct geographic regions in Japan are referred to as the Northern, Central, and Southern regions hereafter) identified via ADMIXTURE and PCA analyses and to detect candidate genes under positive selection in each region. PBS is an *F*_ST_‐based method focusing on genomic regions where distinct allele frequency differentiation has occurred (Yi et al., 2010). The calculations were performed using a custom python script (https://github.com/takuronkym/kpbs) with a set window size of 10 kb. To identify highly differentiated regions, PBS_max_ (Wu et al., 2019), representing the largest PBS value among the three population branches, was calculated. This analysis for PBS_max_ does not require the outgroup as was the case in the study by Montero‐Mendieta et al. (2019). PBS_max_ detects genetic regions that have been selected in only one of them compared with the other two populations. A genomic region (window) included in the upper 0.1% range of the PBS_max_ value distribution was defined as a candidate outlier region for selection. In addition, a null distribution of PBS values for each population was generated using the intergenic (non‐coding) regions for comparison with the PBS_max_ values of the outlier regions. For some outlier regions, the distributions of allele frequencies were visualized using vcftools 0.1.16 (Danecek et al., 2011).

The LFMM was used to examine the relationship between environmental factors and allele frequencies (Frichot et al., 2013). Four environmental factors were selected: temperature (annual average), snowfall (annual maximum depth), precipitation (annual average), and sunlight (annual total hours). These Geographic Information Systems data were retrieved from the website of the Ministry of Land, Infrastructure, Transport, and Tourism of Japan (https://nlftp.mlit.go.jp/ksj/gml/datalist/KsjTmplt‐G02.html). We chose the four environmental factors that vary along a south to north geographic gradient (Figure S1). In addition, these factors were expected to change along a geographical gradient due to the effects of global warming. For LFMM, the number of clusters was fixed at *K* = 3 (number of main clusters in the Japanese population), and only SNPs with false discovery rates below the threshold (<0.01, threshold for precipitation and sunlight; <0.05, threshold for temperature and snowfall since no SNP was detected) were used as candidate SNPs. To avoid losing the selection signal of the candidate SNPs, outlier regions were defined as the flanking 5‐kb upstream and downstream sequences of significantly correlated SNPs (10 kb in total).

For both PBS_max_ and LFMM, genes overlapping with the outlier regions were identified using the GFF model of the reference genome (Yokoi et al., 2018) and defined as candidate genes. The annotations were obtained from the best hits of a blastp search (BLAST 2.7.1+ with the option “‐evalue 1e‐4”) against the *A. mellifera* gene set (GCF_003254395.2_Amel_HAv3.1 from the National Center for Biotechnology Information database). The mutation patterns of SNPs were identified by snpEff 4.5 (Cingolani et al., 2012). Gene ontology (GO) information for *A. cerana* was not directly available at the time of analysis. Thus, GO analysis was performed with PANTHER (Mi et al., 2013; Thomas et al., 2003) using putative homologs of the *Drosophila* gene set (BDGP6 from EnsemblMetazoa).

### 2.6 Comparison between PBS_max_ and LFMM

The genes detected by PBS_max_ could be related to adaptation to a climatic factor in a particular geographic region, such as the Southern or Northern region. If the climatic factor varies along a geographic gradient from south to north, those genes could also be detected via LFMM. Alternatively, the genes detected via PBS_max_ could be related to a local adaptation to a geographic region‐specific environment or nonenvironmental factors. Beckman et al. (2022) investigated whether genes involved in the selection process are related to the gradient of environmental factors by comparing the candidate genes detected via the PBS and LFMM methods. Similarly, we compared the candidate genes detected by the two methods to discuss whether genes subject to selection in each genetically distinct geographic region are related to climate factors along geographic or environmental gradients. For instance, if we detected a candidate gene in the Northern region via PBS_max_, and this gene was also identified as one correlated with temperature by LFMM, this gene, involved in local adaptation in the Northern region, would also be related to temperature along geographic gradients. Conversely, if genes related to local adaptation to geographic region‐specific factors (detected by PBS_max_) are not correlated with environmental variables along a geographic gradient, the local adaptation might prevent from shifting their habitat along the geographic gradient facing environmental changes owing to global warming.

It should be recognized that the methods we applied here have certain weaknesses. For example, the software detecting genes under selection might detect false‐positive or false‐negative genes and use only LFMM and PBS_max_. We further discussed these problems in Section 4.

## 3 RESULTS

### 3.1 Genetic structure and genetic diversity

**FIGURE 2:**
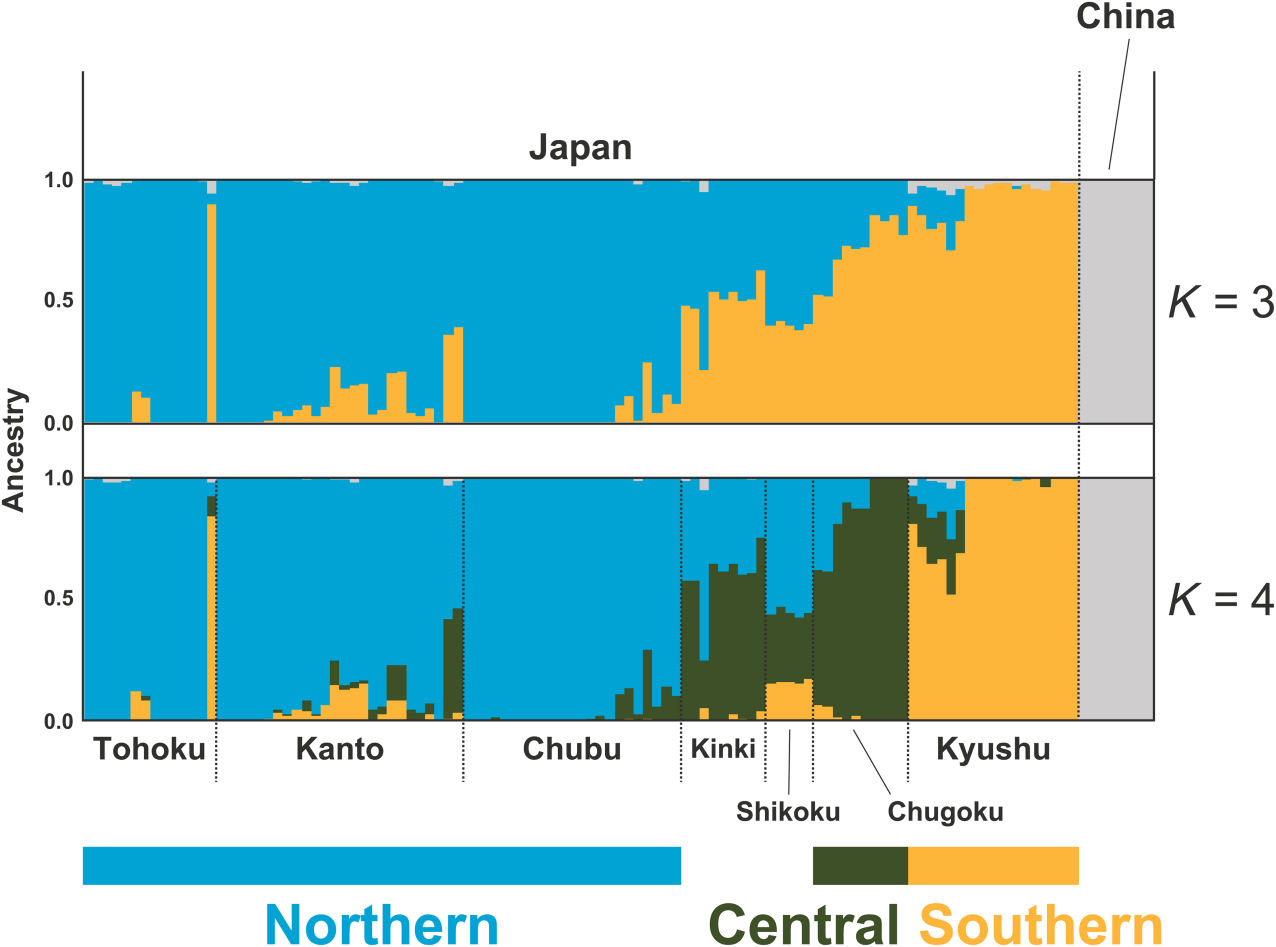
Results of ADMIXTURE analysis of 105 Japanese and eight Chinese samples (*K* = 3 and 4). CV errors with different *K* values are shown in Figure S2.

Approximately 1.27 million SNPs were identified from the whole genome variants of 105 Japanese individuals. Table 1 provides a comprehensive overview of genetic statistics. ADMIXTURE analysis revealed that Japanese samples were distinct from mainland Chinese *A. cerana* (Figure 2; Figures S2 and S3). When including samples from the Korean Peninsula, Korean individuals were considered hybrids between Chinese and Japanese *A. cerana* (Figure S4).

**TABLE 1:** Summary genetic statistics of the *A. c. japonica* populations.

District	Number of samples	(+ putative non‐native)	Polymorphic sites	Nucleotide diversity (PI/10 Kb)
Tohoku	13	1	1,059,958	0.0021
Kanto	24	2	1,178,542	0.0019
Chubu	22	1	1,123,594	0.0018
Kinki	8	1	1,091,490	0.0019
Shikoku	5	0	926,408	0.0018
Chugoku	8	2	990,719	0.0017
Kyushu	17	1	1,160,368	0.0019

**FIGURE 3:**
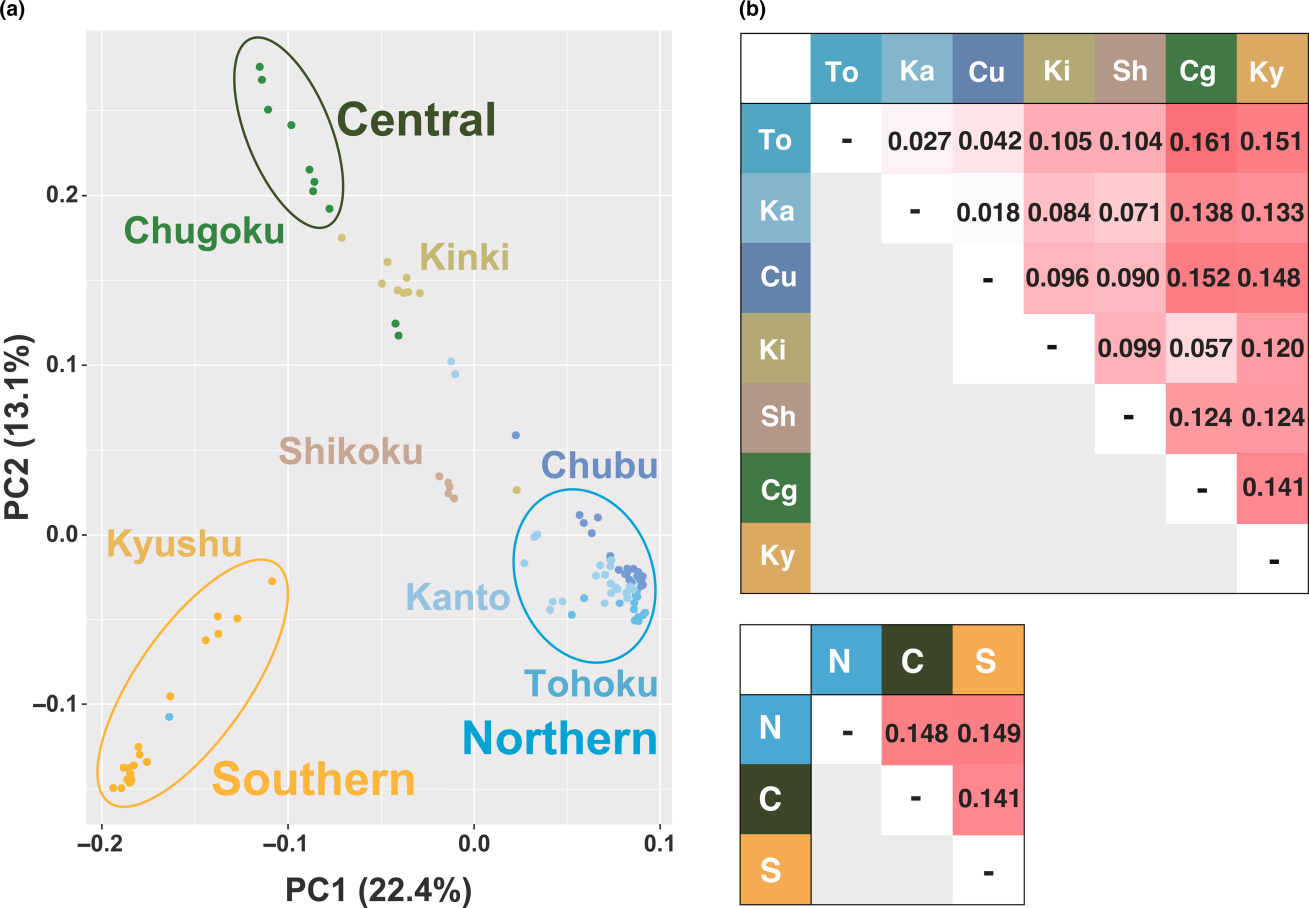
PCA outcomes for 105 Japanese samples (a). Mean *F*_ST_ values between populations (at the level of Districts and Regions) (b). To, Tohoku; Ka, Kanto; Cu, Chubu; Ki, Kinki; Sh, Shikoku; Cg, Chugoku; Ky, Kyushu; N, Northern; C, Central; S, Southern.

Within Japan, the dominant ancestry composition was identified in the Tohoku‐Kanto‐Chubu districts (average 92.7% ~ 97.8%, “blue”), in the Chugoku district (average 92.8%, “green”) and in the Kyushu district (average 91.0%, “orange”) within Japan, and in China at *K* = 4 (“gray,” Figure 2). In some districts, including Kinki (average 37.1% “blue” and 61.8% “green”) and Shikoku (average 55.7% “blue,” 28.3% “green,” and 16.0% “orange”), mixed ancestry was also observed. The results of PCA and mean *F*_ST_ showed that Tohoku, Kanto, and Chubu could be identified as different districts, and Kinki and Shikoku were intermediate between Tohoku‐Kanto‐Chubu and Chugoku or Tohoku‐Kanto‐Chubu, Chugoku, and Kyushu, which were consistent with these genetic structure characteristics (Figure 3). The ADMIXTURE analysis with *K* = 5, indicating genetic differentiation within the Northern region (Figure S3), revealed a slightly lower CV value than *K* = 4. However, CV values at *K* = 4 and *K* = 5 were almost the same (Figure S2), and in addition, the results of PCA and mean *F*_ST_ support the genetic structure with *K* = 4. Thus, we accepted the results with *K* = 4 as the major genetic structure in the subsequent analyses. Consequently, these analyses of the genetic population structure identified three genetically distinct geographic regions (i.e., Northern, Central, and Southern; Figure 2). No notable biases were observed in the number of polymorphic sites and average nucleotide diversity values between geographic regions (Table 1), suggesting that the same level of genetic diversity is maintained throughout Japan.

**FIGURE 4:**
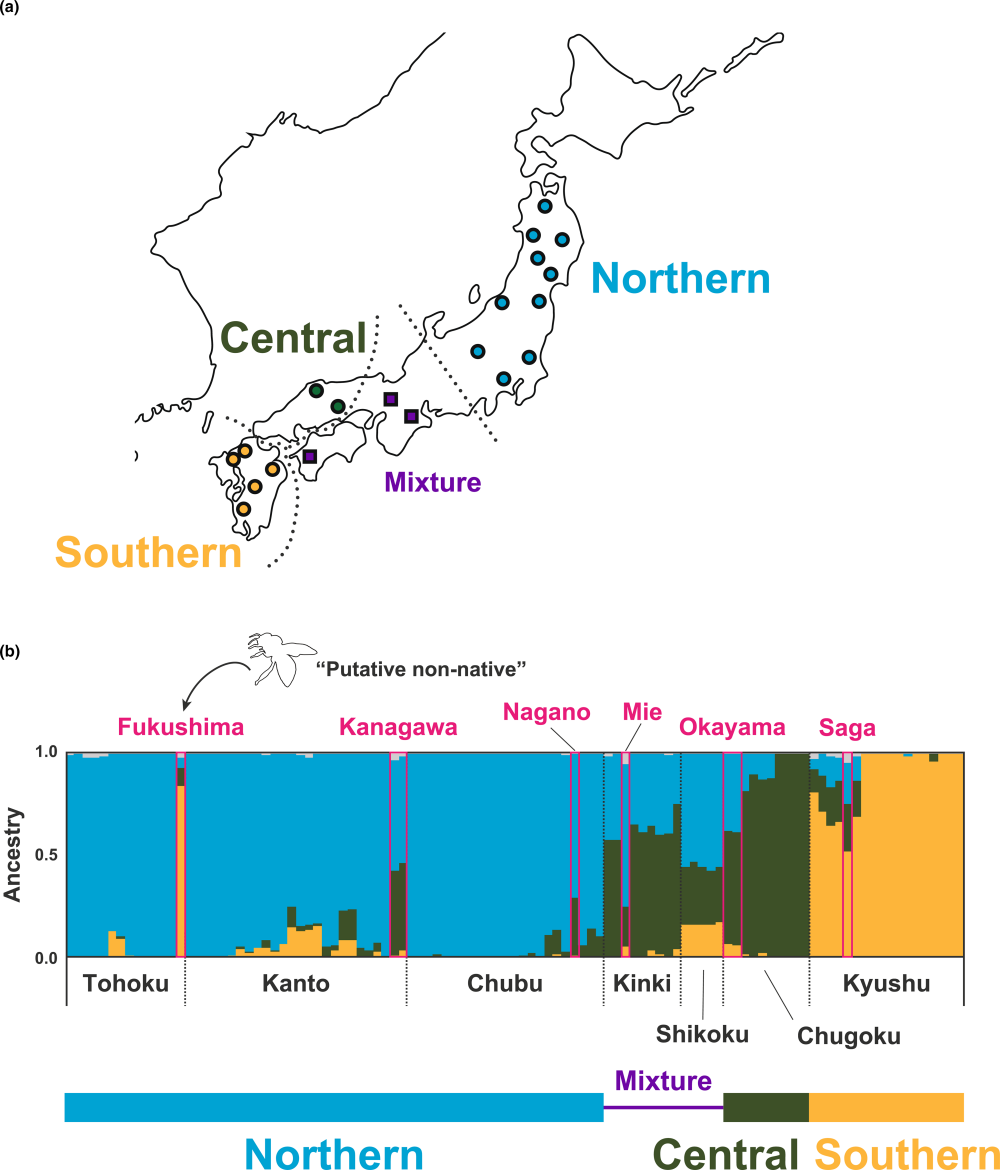
Summary of the genetic structure of Japanese samples (plots in the map of Japan indicate the location of the sampled prefectures) (a). ADMIXTURE results of “putative non‐native” individuals enclosed by pink squares (b). The ADMIXTURE result is the same as that in Figure 2.

In some districts, “putative non‐native” individuals (see Section 2) were found (Figure 4). For example, individuals from Fukushima (Iwaki city) in the Tohoku district shared the same ancestry as that mainly found in the Kyushu district (shown in “orange” in Figure 4), instead of the major ancestry component in Tohoku (shown in “blue”). As a result of interviews with our collaborators regarding the eight “putative non‐native” individuals, one from Fukushima (Iwaki city) and two from Kanagawa (Yokosuka city) were found to be artificially introduced from other districts. In addition, colonies from other areas seemed to be introduced into Nagano (Ueda city), Okayama (Okayama city), and Saga (Saga city). No information about artificial introduction of individuals from Mie (Owase city) was available.

### 3.2 Candidate genes associated with population‐specific divergence

**FIGURE 5:**
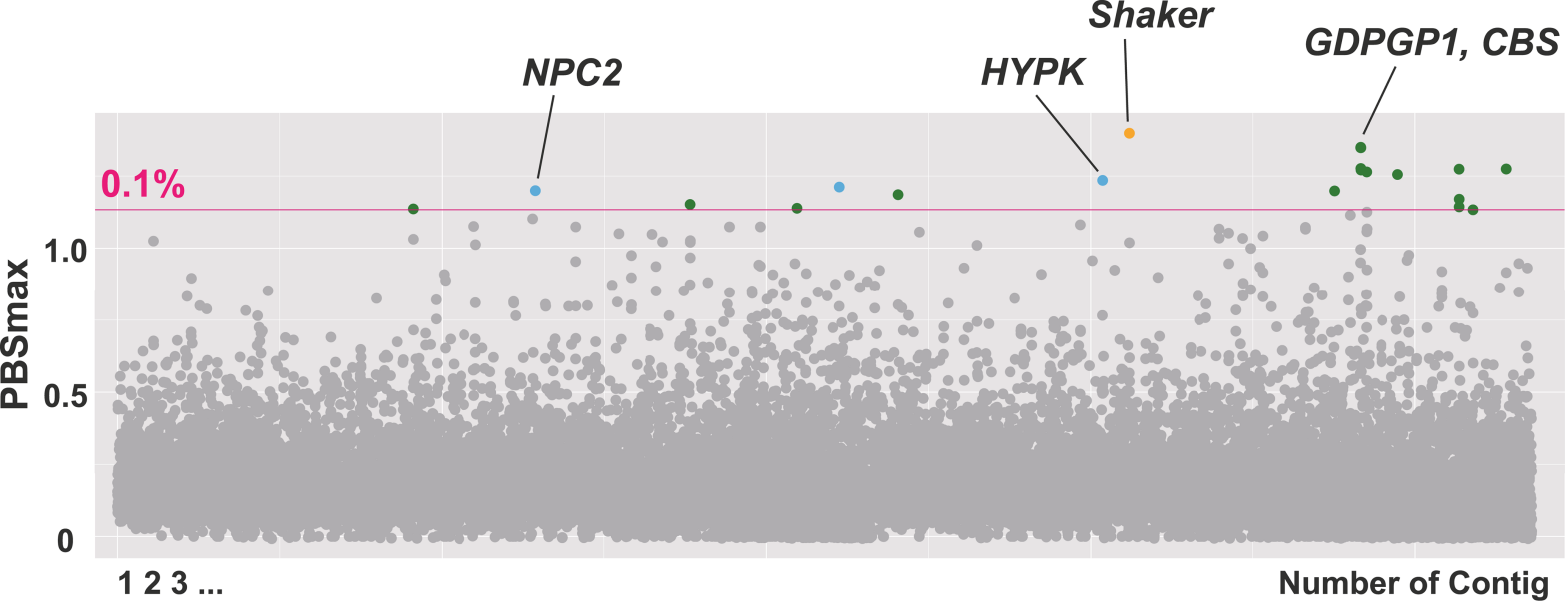
Results of PBS_max_ analysis shown as Manhattan plots. The window size was set at 10 kb. The colors of the outlier plots correspond to each population‐specific divergence (Northern region: blue, Central region: green, and Southern region: orange). The line indicates the threshold of the top 0.1%.

**FIGURE 6:**
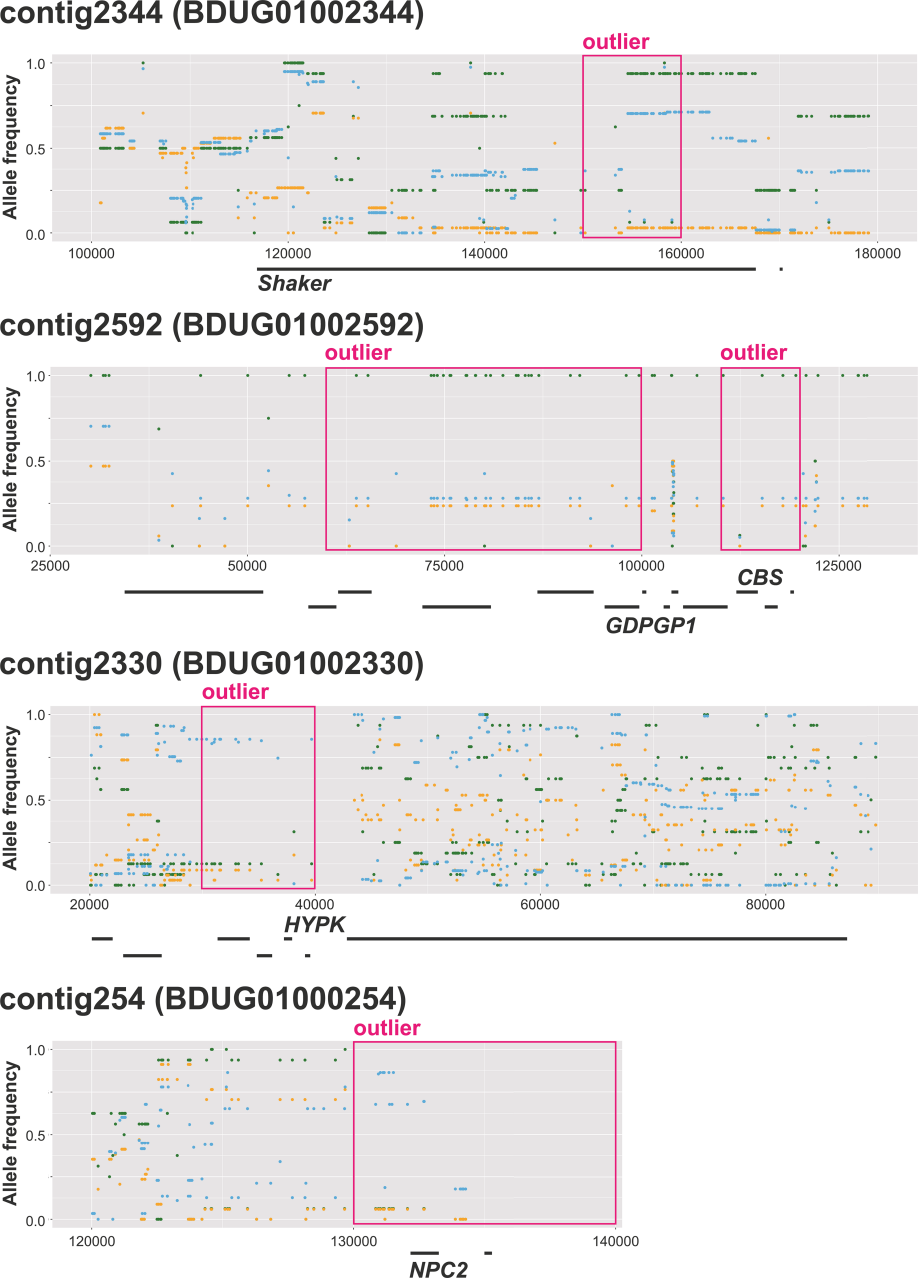
Visualization of the SNP allele frequencies near the PBS_max_ outlier regions. The colors in the plots correspond to each region (Northern region: blue, Central region: green, and Southern region: orange). The boxes indicate the top 0.1% of the outlier windows. The black lines indicate each gene region.

The PBS_max_ analysis showed that all the candidate outlier regions (i.e., upper 0.1% range) fell beyond the threshold (*p* < .01), in which *p*‐values were determined using the null distribution generated from intergenic (non‐coding) regions (Figure 5; Table 2). The 22 detected candidate outlier regions included 25 genes (6, 18, and 1 candidate genes detected in the Northern, Central, and Southern regions, respectively). Among these, seven contained missense mutations that directly affected protein functions (6 and 1 candidate genes in the Central and Southern regions, respectively). To further analyze the details of genetic differentiation among populations, the allele frequencies of the SNPs in outliner regions of some candidate genes were visualized, as shown in Figure 6.

**TABLE 2:** Summary of candidate genes detected by PBS_max_ analysis.

Genomic position	Related windows	Highest PBS_max_ score	Selection	Gene name	Gene ID
contig141 (BDUG01000141): 1–10000	1	1.136	Central	No hit	g2017
contig254 (BDUG01000254): 130001–140000	1	1.200	Northern	NPC intracellular cholesterol transporter 2	g2977
				Estradiol 17‐beta‐dehydrogenase 2	g2978
contig526 (BDUG01000526): 10001–20000	1	1.152	Central	Calcium‐activated potassium channel slowpoke isoform X21	g4299
contig2330 (BDUG01002330): 30001–40000	1	1.235	Northern	WD repeat‐containing protein 91 isoform_X1	g8391
				Transcription initiation protein SPT3 homolog isoform X1	g8392
				Huntingtin‐interacting protein K	g8393
				No hit	g8394
contig2344 (BDUG01002344): 150001–160000	1	1.398	Southern	Potassium voltage‐gated channel protein Shaker isoform X3	g8566
contig2592 (BDUG01002592): 60001–100000, 110001–120000	5	1.350	Central	Probable ATP‐dependent RNA helicase pitchoune	g10995
				Glutathione S‐transferase 1–1	g10996
				Putative thiamine transporter SLC35F3 isoform X7	g10997
				Serine/arginine repetitive matrix protein 1 isoform X1	g10998
				GDP‐D‐glucose phosphorylase 1	g10999
				GRAM domain‐containing protein 1B	g11003
				Cystathionine‐beta‐synthase	g11004
				Trafficking protein particle complex subunit 6B	g11005
				No hit	g11006
contig2603 (BDUG01002603): 40001–50000	1	1.264	Central	Nucleolar complex protein 3 homolog	g11094
				KAT8 regulatory NSL complex subunit 3	g11095
				Phospholipid phosphatase 5 isoform X1	g11096
contig2885 (BDUG01002885): 1–30000	3	1.274	Central	Calpain‐D isoform X1	g12283
				Nucleoporin p58/p45 isoform X1	g12284
				Protein FAM114A2	g12285
contig2942 (BDUG01002942): 1–10000	1	1.133	Central	No hit	g12478

In the candidate outlier region of contig2344 (BDUG01002344), which had the highest PBS_max_ score, *Shaker* (potassium voltage‐gated channel protein Shaker isoform X3), putatively associated with circadian rhythms, was detected as a candidate gene for selection in the Southern region. This gene contained two regional missense mutations with frequencies that diverged among the regions. In contig2592 (BDUG01002592), nine genes were detected from five adjacent scanning windows. These genes showed signals of selection in the Central region and included *GDPGP1* (GDP‐D‐glucose phosphorylase 1) and *CBS* (cystathionine‐beta‐synthase), which are associated with energy metabolism. Among the three candidate outlier regions in the Northern region, six potential candidate genes were detected, including *HYPK* (huntingtin‐interacting protein K) and *NPC2* (NPC intracellular cholesterol transporter 2), which are associated with immune function.

### 3.3 Candidate genes correlated with environmental factors

**FIGURE 7:**
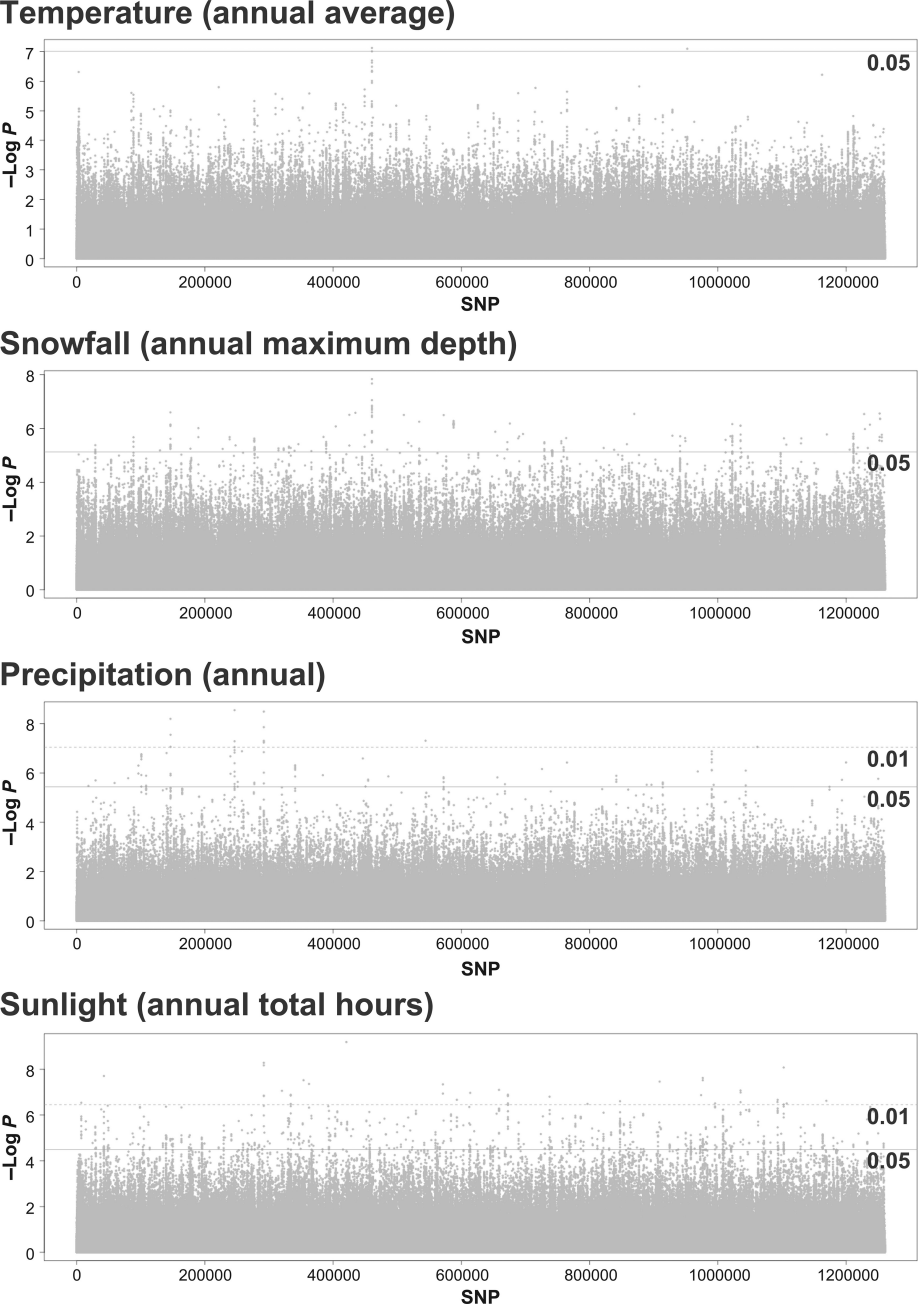
Results of LFMM analysis shown as Manhattan plots. The plots are based on SNP values. The lines indicate the threshold of the false discovery rate 0.05 and 0.01.

The LFMM analysis identified SNPs whose frequencies were significantly correlated with the environmental variables. These SNPs differed depending on the four environmental parameters (Figure 7). About 70 candidate genes overlapping the candidate outlier regions (i.e., the flanking 5‐kb upstream and downstream sequences of the significant SNPs) were detected: 0 associated with temperature, 55 with snowfall, 4 with precipitation, and 14 with sunlight (Table 3; Table S3).

**TABLE 3:** Summary of candidate genes detected by LFMM analysis.

Environmental factor	Related candidate SNP	Contig	FDR	Gene name	Gene ID
Snowfall (annual maximum depth)	17	BDUG01000474	<0.05	Sialin isoform X2	g4091
	8	BDUG01000193	<0.05	MICOS complex subunit Mic60 isoform X3	g2523
	7	BDUG01000108	<0.05	Protein madd‐4 isoform X3	g1664
	7	BDUG01002671	<0.05	Clusterin‐associated protein 1‐like	g11538
	6	BDUG01000042	<0.05	LOW QUALITY PROTEIN: neurogenic locus Notch protein	g897
	5	BDUG01000109	<0.05	Suppressor of lurcher protein 1 isoform X2	g1670
	4	BDUG01000193	<0.05	No hit	g2522
	4	BDUG01002255	<0.05	Neuroligin 3 precursor	g6762
	4	BDUG01002671	<0.05	Uncharacterized protein LOC410375 isoform X1	g11537
	3	BDUG01000020	<0.05	PH domain leucine‐rich repeat‐containing protein phosphatase 2	g555
	3	BDUG01000364	<0.05	Uncharacterized protein LOC100577092	g3587
	3	BDUG01002260	<0.05	No hit	g6914
	2	BDUG01000084	<0.05	Heterogeneous nuclear ribonucleoprotein Q isoform X8	g1399
	2	BDUG01000144	<0.05	Unconventional myosin‐IXb isoform X3	g2033
	2	BDUG01000224	<0.05	Uncharacterized protein LOC100578476	g2773
	2	BDUG01000246	<0.05	Fasciclin‐3 isoform X8	g2909
	2	BDUG01003168	<0.05	No hit	g13031
	2	BDUG01003243	<0.05	Uncharacterized protein LOC107964262	g13156
Precipitation (annual)	5	BDUG01000117	<0.01	Zeta‐sarcoglycan isoform X2	g1796
	3	BDUG01000089	<0.01	Caspase‐1 isoform X3	g1450
Sunlight (annual total hours)	4	BDUG01000017	<0.01	Zeta‐sarcoglycan isoform X2	g1796
	3	BDUG01000146	<0.01	DE‐cadherin isoform X2	g2043
	3	BDUG01000967	<0.01	Uncharacterized protein LOC100578617	g5458
	2	BDUG01002433	<0.01	Protein obstructor‐E	g9671
	2	BDUG01002433	<0.01	Cuticular protein analogous to peritrophins 3‐D precursor	g9672

*Note*: Genes overlapping with two or more outlier regions are shown. Genes overlapping with only one outlier regions are shown in Table S3.

One peak in overlapping outliner regions was detected for temperature and snowfall. However, no gene overlapped with this outlier region. In the snowfall dataset, *Sialin* (sialin isoform X2) was found in 17 overlapping outlier regions. In addition, *NLG‐3* (neuroligin 3 precursor) and *CLUAP1* (clusterin‐associated protein 1‐like), which are associated with nervous system function, were detected. *Obst‐E* (protein obstructor‐E) and *CPAP3‐D* (cuticular protein analogous to peritrophins 3‐D precursor), which are associated with cuticle formation of the exoskeleton, were detected as candidate genes correlated with sunlight. *SGCZ* (zeta‐sarcoglycan isoform X2), which is associated with muscle tissue, was detected as a candidate gene associated with both precipitation and sunlight.

## 4 DISCUSSION

In this study, approximately 1.27 million SNPs were identified by re‐sequencing of 105 individuals from the main Japanese islands, excluding Hokkaido, which has no native *A. c. japonica*. The results of genetic structure analyses indicate that *A. c. japonica* populations in the Northern (Tohoku, Kanto, and Chubu districts), Central (Chugoku district), and Southern (Kyushu district) regions of Japan are genetically divergent (Figures 2 and 3). Subsequently, genome‐wide scans were conducted to detect candidate genes for adaptation by two different approaches. Using population‐specific divergence (by PBS_max_) and correlation with environmental factors (by LFMM), genome‐wide scans were performed to identify candidate genes that may be associated with local adaptation. The detected candidate genes in population‐specific divergence were associated with circadian rhythms, energy metabolism, and immune function (Figures 5 and 6; Table 2). The detected candidate genes concerning environmental factors were associated with the nervous system and skeletal systems (Figure 7; Table 3).

### 4.1 Genetic structure and genetic diversity

The results of this study found genetic differences between the Japanese and continental populations. To date, three major immigration routes among the Japanese islands used by *A. c. japonica* have been proposed such as Tsushima Island, the Ryukyu archipelago (from the Philippines and Taiwan), and Hokkaido (from Sakhalin, Russia). Prior research has indicated that the Tsushima Island route is the most probable scenario (Sasaki, 1999; Takahashi & Yoshida, 2003). It is estimated that the strait between Japan (including Tsushima Island) and the Asian continent was formed approximately 100,000 years ago. Since then, a land bridge between Japan and the Korean Peninsula has not been formed (Ohshima, 1990, 1991). Consequently, it is probable that *A. c. japonica* diverged from the continental *A. cerana* after gene flow from continental populations ceased. Genetic separation of *A. c. japonica* originating in continental *A. cerana* is consistent with the results of whole genome and mitochondrial genome phylogenetic analyses (Ilyasov et al., 2018; Montero‐Mendieta et al., 2019; Okuyama et al., 2017). ADMIXTURE analysis with *K* = 2 (Figure S3) revealed similarities between the reference population and the Western Japanese population (Kyusyu, Chugoku, Shikoku, and Kinki district), indicating that the western population shares a portion of its ancestry with continental *A. cerana*. Additional analyses including samples from the Korean Peninsula (Figure S4), suggest that the Korean Peninsula appears to be a zone of hybridization between Chinese and Japanese samples. Low similarities were observed between the continental and the Northern Japanese populations, so the origins of the Northern population remain unknown. The Northern populations of Japan may have originated from the Northern populations of the continent (e.g., Northeast China and Primorskiy Krai), or it may have evolved independently in Japan. Additional genomic analyses of related unanalyzed populations or public datasets are expected to provide a complete picture of the evolutionary history of *A. c. japonica*.

The results indicated that *A. c. japonica* populations have three genetically diverged geographic regions. Previous studies (Nagamitsu et al., 2016; Takahashi et al., 2007) using partial mitochondrial DNA sequence or some microsatellite loci failed to account for this population genetic structure, suggesting that the fine genetic structure could be only detected by genome‐wide genetic variations. The genetic differentiation of the *A. c. japonica* identified in this study may have been shaped by various factors, including the geological history of the Japanese islands and diverse biomes. PC1 in the PCA analysis and mean *F*_ST_ of *A. c. japonica* (Figure 3) may indicate differentiation reflecting the elongated south–north shape of the Japanese islands, which could be influenced by climatic conditions and/or isolation by distance. Coroian et al. (2014) purported that climate rather than geography separated two European honeybee subspecies in the Carpathian Mountain. In addition, the geological history of the Japanese islands could influence the genetic differences along the south–north axis. The genetic structure of the Genji firefly was bounded by Fossa Magna and islands of Japan (Kato et al., 2020), which is mostly in agreement with the results of this study, indicating that a similar geological history could affect the genetic structure of *A. c. japonica*.

The PC2 axis in PCA indicated that the population of the Chugoku district was uniquely differentiated. Evolutionary features unique to the Chugoku district have been reported in various taxa of plants and animals, owing to the mountain ranges in an east–west direction and the formation of refugia during the last glacial period (Tsurusaki, 2007). Ancient environments in the Chugoku district have likely promoted the genetic differentiation of *A. c. japonica*. The populations of the Kinki and Shikoku districts had a mixture of ancestry composition. In the Kinki and Shikoku districts, hybridization of the ancestries from the Northern, Central, and Southern regions might be responsible for this genetic structure.

### 4.2 Identification of the origin of the samples and “putative non‐native” individuals

The genetic structure of *A. c. japonica* might be affected by the origin of samples used in this study, which were mainly collected from colonies kept by beekeepers. Such colonies of *A. c. japonica* are often derived from local colonies (Fujiwara, 2010) and most of the samples possessed characteristics unique to the local region. However, some individuals might have been introduced from other areas or derived by hybridization between local and introduced populations. In this study, “putative non‐native” individuals were identified by ADMIXTURE analysis of the genetic compositions (Figure 4). The origin of “putative non‐native” individuals from beekeepers was confirmed. Based on interviews with breeders and our collaborators, most of the “putative non‐native” individuals in this analysis had most likely originated from colony introductions. Hence, the genetic structure elucidated in the present study could reflect those of native *A. c. japonica* populations.

The present study suggests that individuals from other districts could be identified by genetic markers. Considering that each local population has a unique genetic structure, introduced colonies are expected to be less fit in non‐native environments. The introduced colonies could also carry pests and pathogens, potentially infecting neighboring populations. For instance, in *A. mellifera* honeybees were infected by parasitic mites and a virus transmitted via introduced honeybees (Wilfert et al., 2016). However, no genetic region to distinguish local populations of *A. c. japonica* has yet been identified (Nagamitsu et al., 2016; Takahashi et al., 2007). The polymorphism information obtained in this study can be used to identify multiple SNPs as potential genomic markers. Once available, SNP markers can be used in both basic and applied studies. In basic studies, SNPs as potential genomic markers can be used to reassess the characteristics of local populations. In applied studies, the information obtained from SNP markers could be applied to identify movement and regional suitability, and the prevention of unwanted genetic introgression between native and introduced populations.

### 4.3 Candidate genes associated with population‐specific divergence

The PBS_max_ analysis detected 22 outlier regions that included 25 candidate genes as those under divergent selection among the three genetically different geographic regions. Among these genes, *Shaker* was detected as the highest signal of the Southern region‐specific gene under selection. *Shaker* encodes a potassium channel potentially associated with abnormal behavior, sleep, and lifespan in *Drosophila* (Cirelli et al., 2005; Salkoff & Wyman, 1981). *Slowpoke* (calcium‐activated potassium channel slowpoke isoform X21) was detected as a candidate gene under selection in the Central region. *Slowpoke* encodes a potassium channel that might be associated with circadian rhythms and physiological activities (https://www.uniprot.org/uniprot/Q03720). Previous studies have also detected potassium channel genes (*Shaw* and *Hcn4*) associated with local adaptations in *A. mellifera* and *A. cerana*. The gene encoding SHAW in honeybees native to the Iberian Peninsula is purportedly related to environmental factors (longitude, cloud cover in April, and precipitation in January; Henriques et al., 2018). *Hcn4* (potassium/sodium hyperpolarization‐activated cyclic nucleotide‐gated channel 4‐like) was identified as a candidate gene associated with high altitude adaptation of the Chinese *A. cerana* (Montero‐Mendieta et al., 2019). These potassium channel genes might be associated with circadian physiological activities and behaviors, which could be related to adaptation to region‐specific trait differences among *A. c. japonica* populations.

Genes associated with energy metabolism in honeybees, such as fat body and lipid storage, could be related to adaptation to local environments (e.g., Chen et al., 2016). In this study, *GDPGP1* was detected as a candidate gene under selection in the Central region, which functions in glycogen metabolism (Singhal et al., 2020). In fact, inhibition of *GDPGP1* indirectly reduced the amount of glycogen in *Caenorhabditis elegans* (Schulz et al., 2020). In honeybees, blood glucose directly provides the energy to the flight muscles but is also stored in the fat body as a form of glycogen (Sasaki, 1997, 1999), as the ability to metabolize glycogen might be important in the winter months. Thus, glycogen‐related functions of *GDPGP1* might be relevant for adaptation to winter temperatures.

Various immune function‐related genes of *A. mellifera* have been associated in local adaptation (e.g., Henriques et al., 2018; Wallberg et al., 2014). For example, *HYPK*, which encodes a chaperone‐like protein associated with the regulation of cell death and apoptosis, and *NPC2*, which encodes a cholesterol transporter that is differentially expressed during disease infection (Doublet et al., 2017; Rittschof et al., 2019), were detected as candidate genes under selection in the Northern region. Both of these genes are related to immune functions, suggesting that immune characteristics might differ between the Northern and other regions.

### 4.4 Candidate genes correlated with environmental factors

In the present study, the allelic frequencies of 55, 4, and 14 candidate genes were correlated with snowfall, precipitation, and sunlight, respectively. In the snowfall dataset, *Sialin* was detected in the outlier regions with high significance. *Sialin* encodes a sialic acid transporter that plays an important role in intracellular homeostasis in lysosomes. Abnormalities in this gene are associated with neurological diseases in humans (e.g., Morin et al., 2004). Notably, sialin‐related genes are expressed in a wide variety of tissues in *Drosophila* (Laridon et al., 2008). *NLG‐3* and *CLUAP1*, both of which are associated with the nervous system, were also detected in the snowfall dataset. In particular, expression of *Nlg‐3* is relatively higher in the order processing centers of the honeybee brain (Biswas et al., 2008). Fuller et al. (2015) found a large genetic differentiation (by means of *F*_ST_) in *Nlg3* between desert and savanna populations of African *A. mellifera*. The genes with allelic frequencies correlated with the snowfall include those associated with functions of the central nervous system.

*SGCZ* was detected as a candidate gene associated with the precipitation and sunlight. *Sgcz* encodes a membrane protein associated with regulation of the nervous and muscle tissues and have been associated with muscular dystrophy in humans (Wheeler et al., 2002). *Obst‐E* and *CPAP3‐D* were identified as candidate genes correlated with sunlight and cuticle formation (Soares et al., 2013; Tajiri et al., 2017). These skeleton functional genes might be associated with protection against dryness and external influences.

None of the candidate genes correlated with temperature, although some significantly correlated genomic regions did not include annotated genes. In addition, the reference genome sequence used in this study (Yokoi et al., 2018) consisted of 3315 contigs and the chromosome structures were not fully reconstructed, implying that peak signals might not have been properly detected.

### 4.5 Relationship between genetic differentiation and climatic factors

The PBS_max_ and LFMM were designed to detect different concepts of selection. The genes detected by PBS_max_ could be related to climatic variables that are the highest in particular geographic regions, such as the Southern and Northern regions, and vary with environmental gradients across the three geographic regions. Those genes could also be detected via LFMM. Alternatively, the genes detected via PBS_max_ could be related to a local adaptation to geographic region‐specific environmental factors. These genes could not be detected by LFMM. Thus, we compared the candidate genes detected by PBS_max_ and LFMM; however, no genes overlapped (Tables 2 and 3). This suggests that genes adapted to specific geographic regions might not associated with genes adapted to climatic factors along geographic gradients. However, our method might not have been able to detect all important candidate genes for adaptation, since all the methods for detecting genes under selection could detect false‐positive or false‐negative genes.

In the LFMM analysis in this study, four environmental factors (temperature snowfall, precipitation, and sunlight) were chosen due to their variation along a south to north geographic gradient. These four variables are major environmental factors expected to change along a geographical gradient owing to the effects of global warming. However, we cannot rule out the existence of other important environmental variables along geographic gradients that could affect honeybees. In addition, only a single method, LFMM was used for detecting genes correlated with environmental gradients. Therefore, we could not detect all important genes having adapted to environmental variables along a geographic gradient. Different methods might be able to detect distinct regions under selection due to varying abilities to identify ongoing selection. However, the common genes detected by different methods are considered to show stronger correlations. Therefore, even if certain genes remained undetected by LFMM, they might not strongly correlate with the environmental variables. Nevertheless, since we did not include all environments and used only LFMM, we cannot conclude with all certainty that genes involved in local adaptation would not be related to the adaptation to environmental gradients.

*Shaker*, which is associated with circadian rhythms, and *GDPGP1*, which is associated with energy metabolism, could be related to climatic factors such as temperature and sunlight. However, *GDPGP1* was identified as a gene adapted to the Central region. In *Shaker*, the allele frequencies of SNPs identified as outliers do not differ between the Southern and Northern regions (Figure 6). These results also support that there is no association between genes adapted to specific regions and climatic factors along geographic or environmental gradients. Under climatic changes such as global warming, local adaptations related to the genes detected in our study could be a contributing factor to disturbing shifting habitats in Japanese honeybee populations.

## 5 CONCLUSIONS

In this study, genetic structure and local adaptation of *A. c. japonica* were examined by re‐sequencing the genomes of 105 individuals from the three main islands of Japan. The results demonstrated that this subspecies is genetically distinct from the mainland Chinese *A. cerana*. Added to this, *A. c. japonica* populations in the Northern (Tohoku‐Kanto‐Chubu districts), Central (Chugoku district), and Southern (Kyushu district) regions of Japan had diverged genetically. We could identify “putative non‐native” individuals introduced from other geographic regions. Genome‐wide scans were performed to identify candidate genes for adaptation using two distinct methods: those associated with the geographically distinct region‐specific adaptation and those related to climatic environmental factors along the geographic gradient. However, no candidate genes detected via the PBS_max_ and LFMM methods overlapped. The present results imply that candidate genes detected as local adaptation to genetically distinct populations may not be associated with climatic factors along simple north–south geographic gradients. However, the present study did not consider all important environmental variables and used limited methods for detecting candidate genes. Thus, more robust methods are required for examining the role of local adaptation under global warming.

## AUTHOR CONTRIBUTIONS

**Takeshi Wakamiya:** Conceptualization (lead); data curation (lead); formal analysis (lead); funding acquisition (lead); methodology (lead); project administration (equal); software (lead); visualization (lead); writing – original draft (lead); writing – review and editing (lead). **Takahiro Kamioka:** Data curation (supporting); formal analysis (supporting); methodology (supporting). **Yuu Ishii:** Data curation (supporting); formal analysis (supporting); methodology (supporting). **Jun‐ichi Takahashi:** Resources (equal). **Taro Maeda:** Investigation (supporting); resources (lead). **Masakado Kawata:** Conceptualization (lead); formal analysis (supporting); funding acquisition (lead); methodology (supporting); project administration (lead); resources (lead); supervision (lead); visualization (supporting); writing – original draft (supporting); writing – review and editing (lead).

## CONFLICT OF INTEREST STATEMENT

The authors declare no conflicts of interest for this work.

## Supporting information


Data S1:

## Data Availability

The original NGS reads registered to the DNA Data Bank of Japan (DDBJ) database under BioProject PRJDB14080 (DRR397616–DRR397720). Python script for PBS analysis is available at https://github.com/takuronkym/kpbs.
