# Supplementary material for: Genetic differentiation and local adaptation of the Japanese honeybee, Apis cerana japonica
Source: Ecol Evol. 2023 Sep 29;13(10):e10573. doi: 10.1002/ece3.10573 (PMC10541296; doi:10.1002/ece3.10573)

Supporting/Supplemental Information

Genetic differentiation and local adaptation of the Japanese honeybee, *Apis cerana japonica*

Takeshi Wakamiya[1,2], Takahiro Kamioka[1], Yuu Ishii[1], Jun-ichi Takahashi[3], Taro Maeda[4], Masakado Kawata[1]

1 Graduate School of Life Sciences, Tohoku University, Sendai, Japan
2 Department of Biological Sciences, Tokyo Metropolitan University, Hachioji, Japan
3 Faculty of Life Sciences, Kyoto Sangyo University, Kyoto, Japan
4 Institute for Agro-Environmental Sciences (NIAES), NARO, Tsukuba, Japan

Corresponding author
Takeshi Wakamiya, takeshi.waka38@gmail.com (waka38t@tmu.ac.jp)
Masakado Kawata, kawata@tohoku.ac.jp

A)

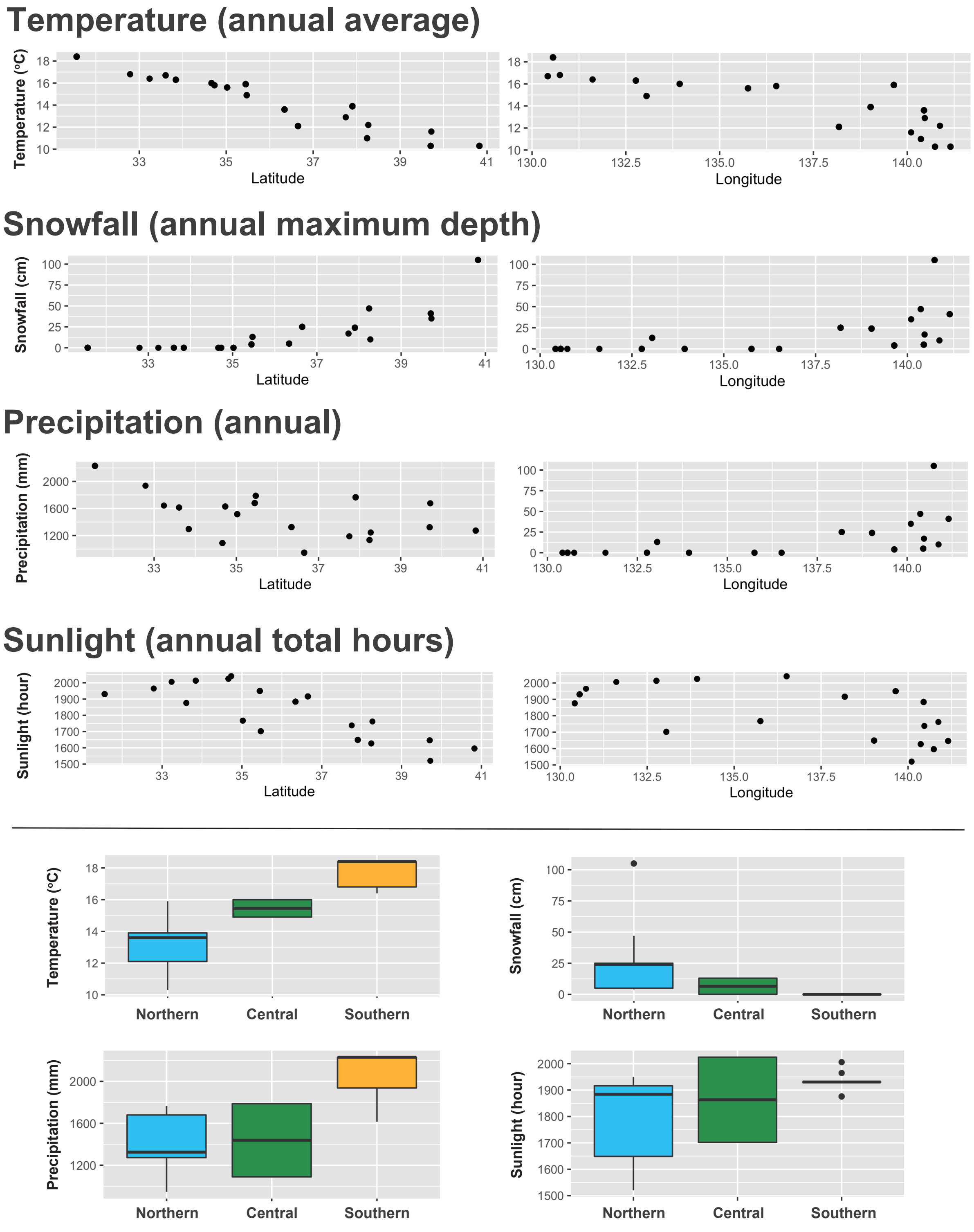
Temperature (annual average)
Temperature (°C)
Latitude
Longitude
Snowfall (annual maximum depth)
Snowfall (cm)
Latitude
Longitude
Precipitation (annual)
Precipitation (mm)
Latitude
Longitude
Sunlight (annual total hours)
Sunlight (hour)
Latitude
Longitude
Northern
Central
Southern

B)

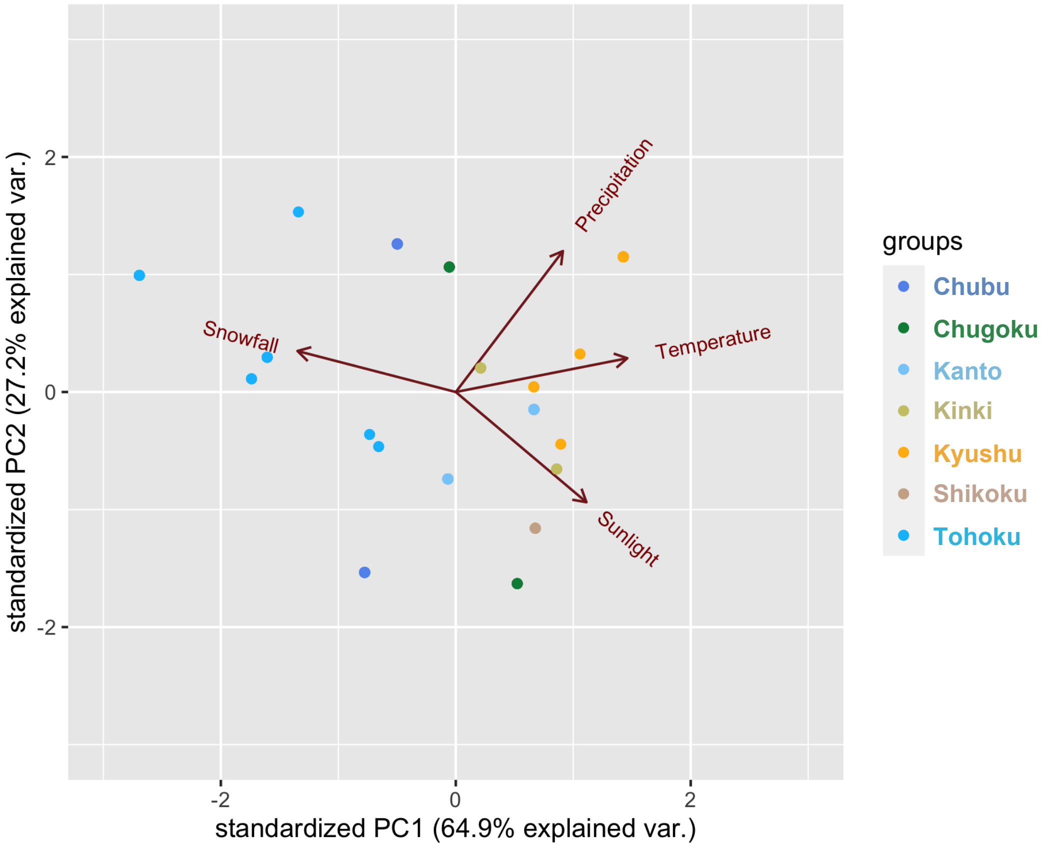

Figure S1. Four climatic environmental variables along the geographic regions (A) and the outcomes of Principle Component Analysis (PCA) for sampling regions and four climatic environmental variables (B). PC1 and PC2 explain 64.9% and 27.2% of the variance, respectively. The arrows indicate the biplots of the four climatic environmental variables. PC1 and PC2 reflect changes in temperatures and snowfall along a north to South gradient and those in the precipitation and sunlight factors along the gradient from the Sea of Japan to the Pacific Ocean, respectively.

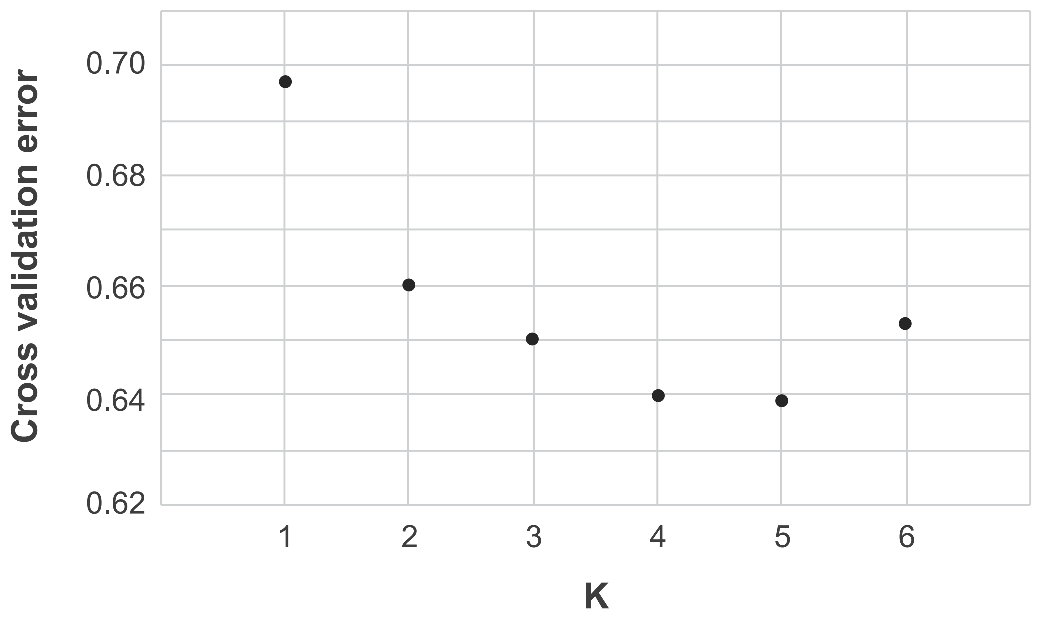

Figure S2. CV errors with different K values in the ADMIXTURE analysis of 105 Japanese and eight Chinese samples.

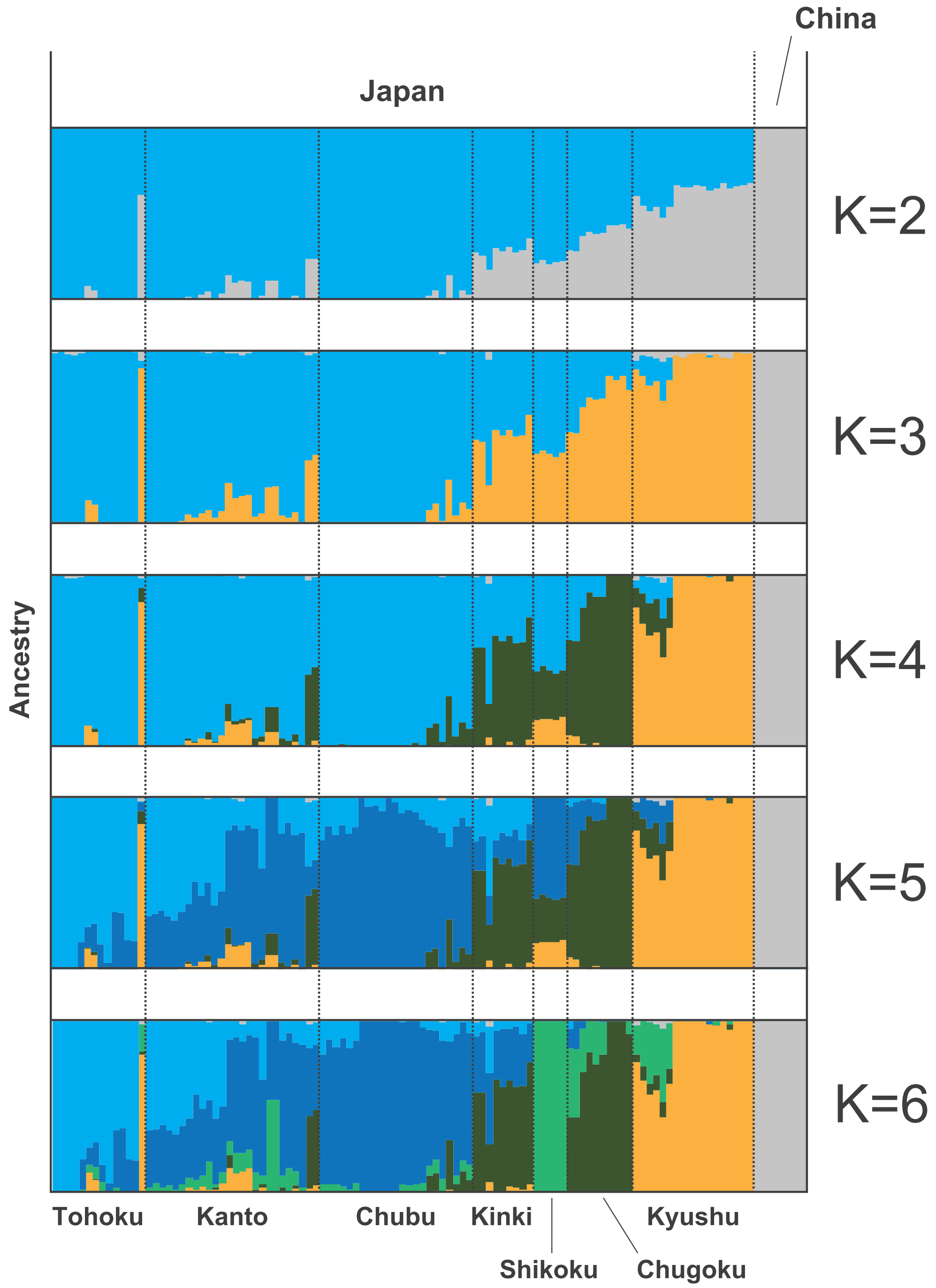

Figure S3. The results of ADMIXTURE analysis of 105 Japanese and 8 Chinese samples with different K values (K = 2 to 6).

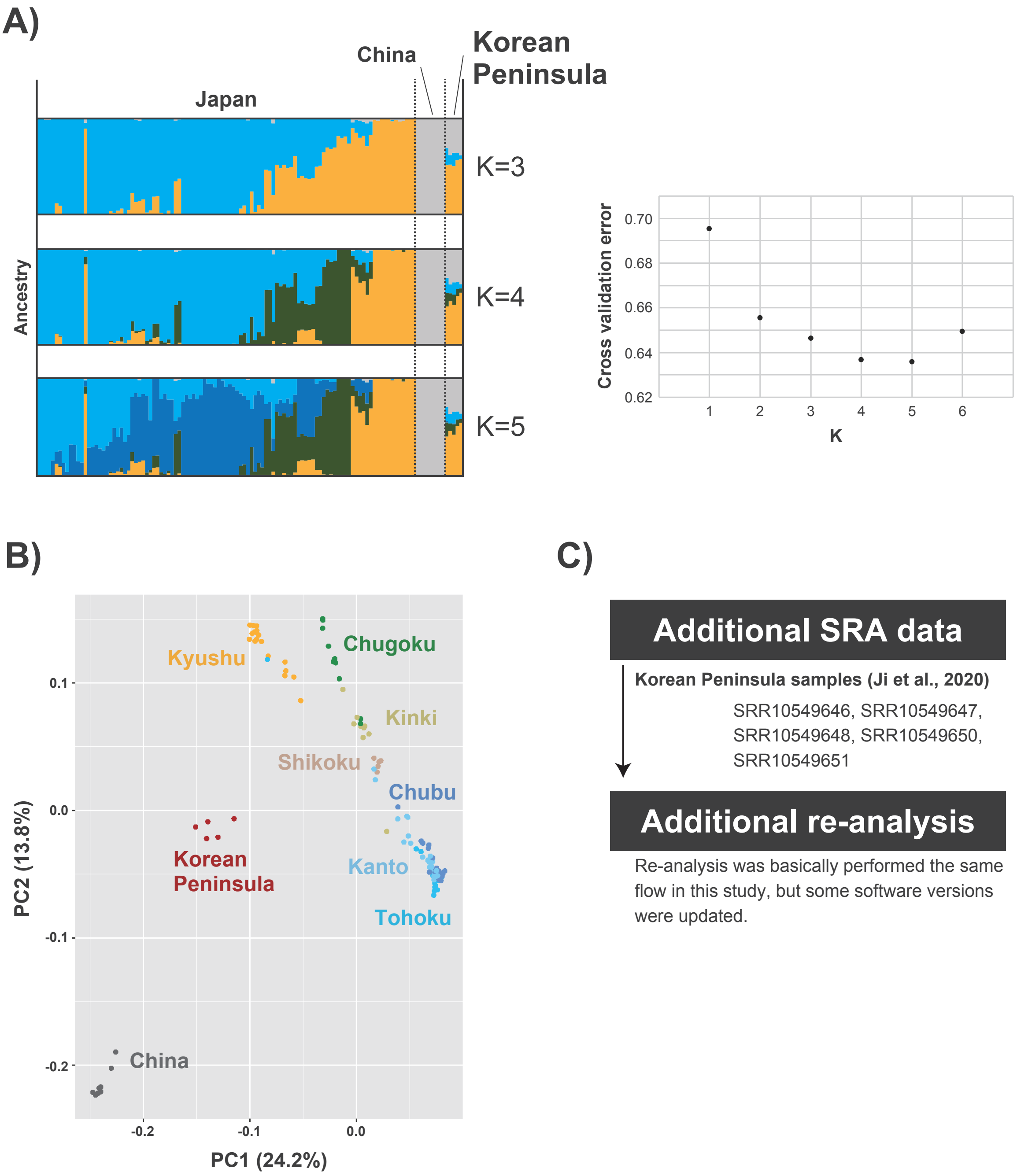

Figure S4. Additional ADMIXTURE re-analysis, including Korean Peninsula samples (A). Additional PCA re-analysis, including Korean Peninsula samples (B). Details of public SRA data used in re-analysis (C).

Table S1. Summary of *A. c. japonica* samples.

District	Prefecture	Sample name (Area + Number)	Year	Collection	ID	Putative non-native
Tohoku	Aomori	Mutsu	2015	NARO	B_index13	
	Aomori	Hirosaki1	2015	Other	A_index14	
	Aomori	Hirosaki2	2015	Other	B_index1	
	Aomori	Hirosaki3	2015	Other	B_index2	
	Akita	Yurihonjyo	2015	NARO	B_index3	
	Iwate	Morioka1	2017	Other	A_index1	
	Iwate	Morioka2	2017	Other	A_index2	
	Iwate	Morioka3	2017	Other	A_index3	
	Iwate	Morioka4	2017	Other	A_index4	
	Yamagata	Yamagata1	2015	NARO	B_index7	
	Yamagata	Yamagata2	2015	NARO	B_index8	
	Miyagi	Kakuda	2015	NARO	B_index9	
	Fukushima	Fukushima	2015	NARO	B_index10	
	Fukushima	Iwaki	2015	NARO	B_index14	Yes
Kanto	Ibaraki	Tokaimura	2017	NARO	C_index12	
	Ibaraki	Naka	2017	NARO	G_index23	
	Ibaraki	Kasama	2016	NARO	F_index23	
	Ibaraki	Omitama	2014	NARO	G_index6	
	Ibaraki	Hokota	2016	NARO	F_index10	
	Ibaraki	Kashima	2017	NARO	C_index7	
	Ibaraki	Itako	2015	NARO	E_index10	
	Ibaraki	Kasumi	2015	NARO	D_index12	
	Ibaraki	Tsuchiura	2014	NARO	G_index13	
	Ibaraki	Tsukubamirai	2017	NARO	D_index1	
	Ibaraki	Joso	2017	NARO	C_index5	
	Ibaraki	Bando	2015	NARO	D_index10	
	Kanagawa	Yokohama1	2014	NARO	G_index12	
	Kanagawa	Yokohama2	2015	NARO	D_index9	
	Kanagawa	Yokohama3	2014	NARO	E_index3	
	Kanagawa	Yokohama4	2014	NARO	E_index5	
	Kanagawa	Yokohama5	2014	NARO	D_index13	
	Kanagawa	Yokohama6	2014	NARO	D_index14	
	Kanagawa	Sagamihara1	2014	NARO	G_index9	
	Kanagawa	Sagamihara2	2014	NARO	E_index7	
	Kanagawa	Sagamihara3	2014	NARO	G_index11	
	Kanagawa	Sagamihara4	2014	NARO	E_index6	
	Kanagawa	Sagamihara5	2014	NARO	G_index10	
	Kanagawa	Yokosuka1	2014	NARO	E_index2	
	Kanagawa	Yokosuka2	2014	NARO	E_index1	Yes
	Kanagawa	Yokosuka3	2014	NARO	G_index8	Yes

(Table S1.)

District	Prefecture	Sample name (Area + Number)	Year	Collection	ID	Putative non-native
Chubu	Niigata	Niigata	2018	NARO	C_index11	
	Niigata	Minamikambaragun_Tagamimachi1	2015	NARO	D_index7	
	Niigata	Minamikambaragun_Tagamimachi2	2015	NARO	D_index8	
	Niigata	Minamikambaragun_Tagamimachi3	2015	NARO	D_index2	
	Niigata	Minamikambaragun_Tagamimachi4	2015	NARO	D_index3	
	Niigata	Minamikambaragun_Tagamimachi5	2015	NARO	D_index4	
	Niigata	Minamiuonuma1	2014	NARO	F_index5	
	Niigata	Minamiuonuma2	2014	NARO	F_index6	
	Niigata	Minamiuonuma3	2016	NARO	F_index8	
	Niigata	Minamiuonuma4	2016	NARO	F_index9	
	Niigata	Minamiuonuma5	2015	NARO	D_index11	
	Niigata	Minamiuonuma6	2014	NARO	D_index23	
	Nagano	Saku	2017	NARO	C_index3	
	Nagano	Omachi	2016	NARO	F_index7	
	Nagano	Matsumoto	2017	NARO	C_index8	
	Nagano	Kamiminochigun_Ogawamura	2015	NARO	D_index5	
	Nagano	Kamiinagun_Miyadamura	2017	NARO	C_index23	
	Nagano	Kamiinagun_Tatsunomachi	2016	NARO	G_index4	
	Nagano	Shimoinagun_Takamorimachi	2014	NARO	E_index4	
	Nagano	Ueda	2016	NARO	E_index8	Yes
	Nagano	Suwagun_Haramura	2014	NARO	G_index7	
	Nagano	Komagane	2017	NARO	C_index1	
	Nagano	Iida	2015	NARO	F_index2	
Kinki	Mie	Suzuka	2016	NARO	F_index4	
	Mie	Tsu	2015	NARO	F_index1	
	Mie	Owase	2016	NARO	G_index3	Yes
	Mie	Nabari	2016	NARO	G_index2	
	Kyoto	Kyoto1	2016	NARO	F_index11	
	Kyoto	Kyoto2	2016	NARO	F_index12	
	Kyoto	Kyoto3	2016	NARO	F_index13	
	Kyoto	Kyoto4	2016	NARO	F_index14	
	Kyoto	Kyoto5	2015	NARO	E_index11	
Shikoku	Ehime	Toon1	2017	NARO	C_index2	
	Ehime	Toon2	2017	NARO	C_index13	
	Ehime	Toon3	2015	NARO	D_index6	
	Ehime	Toon4	2015	NARO	E_index9	
	Ehime	Toon5	2013	NARO	G_index14	

(Table S1.)

District	Prefecture	Sample name (Area + Number)	Year	Collection	ID	Putative non-native
Chugoku	Okayama	Okayama1	2015	NARO	G_index5	Yes
	Okayama	Okayama2	2015	NARO	E_index23	Yes
	Okayama	Okayama3	2015	NARO	E_index12	
	Okayama	Okayama4	2015	NARO	E_index13	
	Okayama	Okayama5	2015	NARO	E_index14	
	Okayama	Okayama6	2015	NARO	F_index3	
	Shimane	Matsue1	2017	NARO	C_index4	
	Shimane	Matsue2	2017	NARO	C_index9	
	Shimane	Matsue3	2017	NARO	C_index10	
	Shimane	Matsue4	2017	NARO	C_index14	
Kyushu	Oita	Yufu	2015	NARO	B_index6	
	Fukuoka	Kurume1	2017	NARO	C_index6	
	Fukuoka	Kurume2	2016	NARO	G_index1	
	Fukuoka	Onojyo	2015	NARO	B_index5	
	Saga	Saga	2015	NARO	B_index12	Yes
	Kumamoto	Amakusa	2015	NARO	B_index4	
	Kagoshima	Kirishima1	2017	Other	A_index5	
	Kagoshima	Kirishima2	2017	Other	A_index6	
	Kagoshima	Kirishima3	2017	Other	A_index7	
	Kagoshima	Kirishima4	2017	Other	A_index8	
	Kagoshima	Kirishima5	2017	Other	A_index9	
	Kagoshima	Kirishima6	2017	Other	A_index10	
	Kagoshima	Kirishima7	2017	Other	A_index20	
	Kagoshima	Kirishima8	2017	Other	A_index23	
	Kagoshima	Kirishima9	2015	NARO	B_index11	
	Kagoshima	Minamikyushu1	2015	Other	A_index11	
	Kagoshima	Minamikyushu2	2015	Other	A_index12	
	Kagoshima	Minamikyushu3	2015	Other	A_index13	

Table S2. Summary of Chinese samples from the Sequence Read Archive database.

Country	Region	Accession number	ID	Refarence
China	QY	SRR6301338	QY04	Chen et al. 2018
	QY	SRR6301340	QY06	Chen et al. 2018
	QY	SRR6301325	QY08	Chen et al. 2018
	QY	SRR6301364	QY09	Chen et al. 2018
	YL	SRR6301393	YL01	Chen et al. 2018
	YL	SRR6301391	YL03	Chen et al. 2018
	YL	SRR6301390	YL04	Chen et al. 2018
	YL	SRR6301385	YL09	Chen et al. 2018

Table S3. Summary of candidate genes detected by LFMM analysis. Genes overlapping with only one outlier regions are shown.

Environmental factor	Related candidate SNP	Contig	FDR	Gene name	gene ID
Precipitation (annual)	1	BDUG01002393	<0.01	proclotting enzyme	g9238
	1	BDUG01002393	<0.01	atrial natriuretic peptide receptor 1 isoform X1	g9239
Sunlight (annual total hours)	1	BDUG01000485	<0.01	uncharacterized protein LOC725241 isoform X2	g4137
	1	BDUG01000485	<0.01	uncharacterized protein LOC725241 isoform X2	g4138
	1	BDUG01000549	<0.01	collagen alpha chain CG42342 isoform X12	g4386
	1	BDUG01000799	<0.01	serine protease 53	g5133
	1	BDUG01000799	<0.01	trypsin-7-like	g5134
	1	BDUG01002256	<0.01	uncharacterized protein LOC409805 isoform X2	g6804
	1	BDUG01002527	<0.01	SLIT-ROBO Rho GTPase-activating protein 1 isoform X4	g10523
	1	BDUG01002308	<0.01	No hit	g8020
	1	BDUG01002336	<0.01	No hit	g8485
Snowfall (annual maximum depth)	1	BDUG01000055	<0.05	uncharacterized protein LOC100577283 isoform X2	g1041
	1	BDUG01000079	<0.05	No hit	g1350
	1	BDUG01000146	<0.05	DE-cadherin isoform X2	g2043
	1	BDUG01000155	<0.05	discoidin domain-containing receptor 2	g2149
	1	BDUG01000173	<0.05	cGMP-specific 3',5'-cyclic phosphodiesterase isoform X1	g2355
	1	BDUG01000205	<0.05	No hit	g2636
	1	BDUG01000290	<0.05	No hit	g3201
	1	BDUG01000338	<0.05	aryl hydrocarbon receptor protein 1 isoform X2	g3498
	1	BDUG01000436	<0.05	No hit	g3933
	1	BDUG01000741	<0.05	transcription initiation factor TFIID subunit 3-like isoform X5	g4992
	1	BDUG01001024	<0.05	glycine-rich cuticle protein	g5563
	1	BDUG01001476	<0.05	serine/threonine-protein kinase Warts	g6136
	1	BDUG01002010	<0.05	cytochrome P450 302a1, mitochondrial	g6451
	1	BDUG01002257	<0.05	uncharacterized protein LOC408844 isoform X1	g6817
	1	BDUG01002257	<0.05	uncharacterized protein LOC408844 isoform X1	g6818
	1	BDUG01002261	<0.05	quinone oxidoreductase-like protein 2-like	g6955
	1	BDUG01002273	<0.05	synaptotagmin 1 isoform X1	g7288
	1	BDUG01002321	<0.05	mediator of RNA polymerase II transcription subunit 4	g8246
	1	BDUG01002321	<0.05	SAC3 domain-containing protein 1	g8247
	1	BDUG01002321	<0.05	laccase-1	g8248
	1	BDUG01002325	<0.05	No hit	g8341
	1	BDUG01002359	<0.05	glucose dehydrogenase [FAD, quinone] isoform X1	g8789
	1	BDUG01002359	<0.05	glucose oxidase	g8790
	1	BDUG01002433	<0.05	No hit	g9679
	1	BDUG01002460	<0.05	No hit	g9925
	1	BDUG01002461	<0.05	CUGBP Elav-like family member 4 isoform X14	g9926
	1	BDUG01002671	<0.05	peptidyl-tRNA hydrolase ICT1, mitochondrial	g11539
	1	BDUG01002737	<0.05	microphthalmia-associated transcription factor isoform X1	g11835
	1	BDUG01002880	<0.05	No hit	g12268
	1	BDUG01002880	<0.05	No hit	g12269
	1	BDUG01002929	<0.05	putative hydroxypyruvate isomerase	g12442
	1	BDUG01002963	<0.05	suppressor of variegation 3-9 isoform 1	g12546
	1	BDUG01002963	<0.05	dihydroxyacetone phosphate acyltransferase	g12547
	1	BDUG01003133	<0.05	uncharacterized protein MAL13P1.304 isoform X1	g12951
	1	BDUG01003243	<0.05	phenylalanine--tRNA ligase beta subunit isoform X1	g13153
	1	BDUG01003243	<0.05	ankyrin repeat domain-containing protein 17 isoform X1	g13154
	1	BDUG01003243	<0.05	NAD-dependent protein deacetylase sirtuin-1 isoform X2	g13155

Supplementary text 1. Commands and parameters for Quality control, Variant calling, and SNP calling. Custom scripts are available on request to Takeshi Wakamiya (takeshi.waka38@gmail.com).

Quality control

Repeat

Fastx-toolkit (http://hannonlab.cshl.edu/fastx_toolkit/)

```
$ fastq_quality_filter -v -Q 33 -q 30 -p 50 -i {sample ID}_R1.fastq -o FQ_{sample ID}_R1.fastq
$ fastq_quality_filter -v -Q 33 -q 30 -p 50 -i {sample ID}_R2.fastq -o FQ_{sample ID}_R2.fastq
```

Custom script 1 | Output only paired reads.

$ perl **output_only_paired_reads.pl** FQ_{sample ID}_R1.fastq FQ_{sample ID}_R2.fastq qc_FQ_{sample ID}_R1.fastq qc_FQ_{sample ID}_R2.fastq

Variant calling

Index | Acj_ref_genome: *Apis cerana japonica* reference genome sequence (Yokoi et al., 2018).

```
$ bwa index {Acj_ref_genome}.fasta
```

Mapping | BWA-MEM and SAMtools1.8

```
$ bwa mem {Acj_ref_genome}.fasta qc_FQ_{sample ID}_R1.fastq qc_FQ_{sample ID}_R2.fastq > {sample ID}.sam
$ samtools view -bS {sample ID}.sam > {sample ID}.bam
$ samtools sort {sample ID}.bam -o {sample ID}_s.bam
$ samtools index {sample ID}_s.bam
$ samtools rmdup {sample ID}_s.bam {sample ID}_sr.bam
$ samtools index {sample ID}_sr.bam
```

Repeat

Index 2

```
$ samtools faidx {Acj_ref_genome}.fasta
```

Variant calling | SAMtools1.8 and BCFtools1.8

```
$ samtools mpileup -uf {Acj_ref_genome}.fasta {sample ID}_sr.bam | bcftools call -O b -v -c -> {sample ID}sr.bcf
$ bcftools view {sample ID}sr.bcf | vcfutils.pl varFilter -d 5 -D 100 > {sample ID}sr.vcf
```

Prepere for merge

```
$ bgzip {sample ID}sr.vcf
$ tabix -p vcf {sample ID}sr.vcf.gz
```

Repeat

Merge | vcftools 0.1.16

```
$ vcf-merge -R 0/0 {sample ID}sr.vcf.gz {sample ID}sr.vcf.gz {sample ID}sr.vcf.gz ... | bgzip -c > {vcf_name}.gz
```

Custom script 2, 3 and 4 | Pre-filtering the vcf file (delite INDEL variants, add variant IDs, sort by position numbers).

$ sh **make_set_vcf_v3.sh** {vcf_name}.vcf

$ bash **plink_add_snp_id.sh** {vcf_name}_kansei.vcf

$ sh **vcf_sort_v3.sh** {vcf_name}_kansei_addid.vcf

SNP filtering

filtering for genetic structure analyses (minor allele frequency and LD removal step) | PLINK 1.90

$ plink --vcf {vcf_name}_kansei_addid_sorted.vcf --allow-extra-chr --maf 0.05 --make-bed -out maf5_{bfile_name}

$ plink --bfile maf5_{output_name} --allow-extra-chr --indep-pairwise 50 10 0.1

$ plink --bfile maf5_{output_name} --allow-extra-chr --extract plink.prune.in --make-bed --out pru_maf5_{bfile_name}

filtering for ADMIXTURE analysis (The chromosome nubers were set to “0” .)

$ awk '{$1=0;print $0}' pru_maf5_{bfile_name}.bim > pru_maf5_{bfile_name}.bim.tmplete

Then, delete original “pru_maf5_{bfile_name}.bim” , and
rename “pru_maf5_{bfile_name}.bim.tmplete” to “pru_maf5_{bfile_name}.bim” .

filtering for normal analyses (minor allele frequency) | PLINK 1.90

$ vcftools --vcf {vcf_name}_kansei_addid_sorted.vcf --maf 0.05 --out maf5_{norm_vcf_name} --recode

Supplementary text 2. The Bioinformatics workflow used in this study.

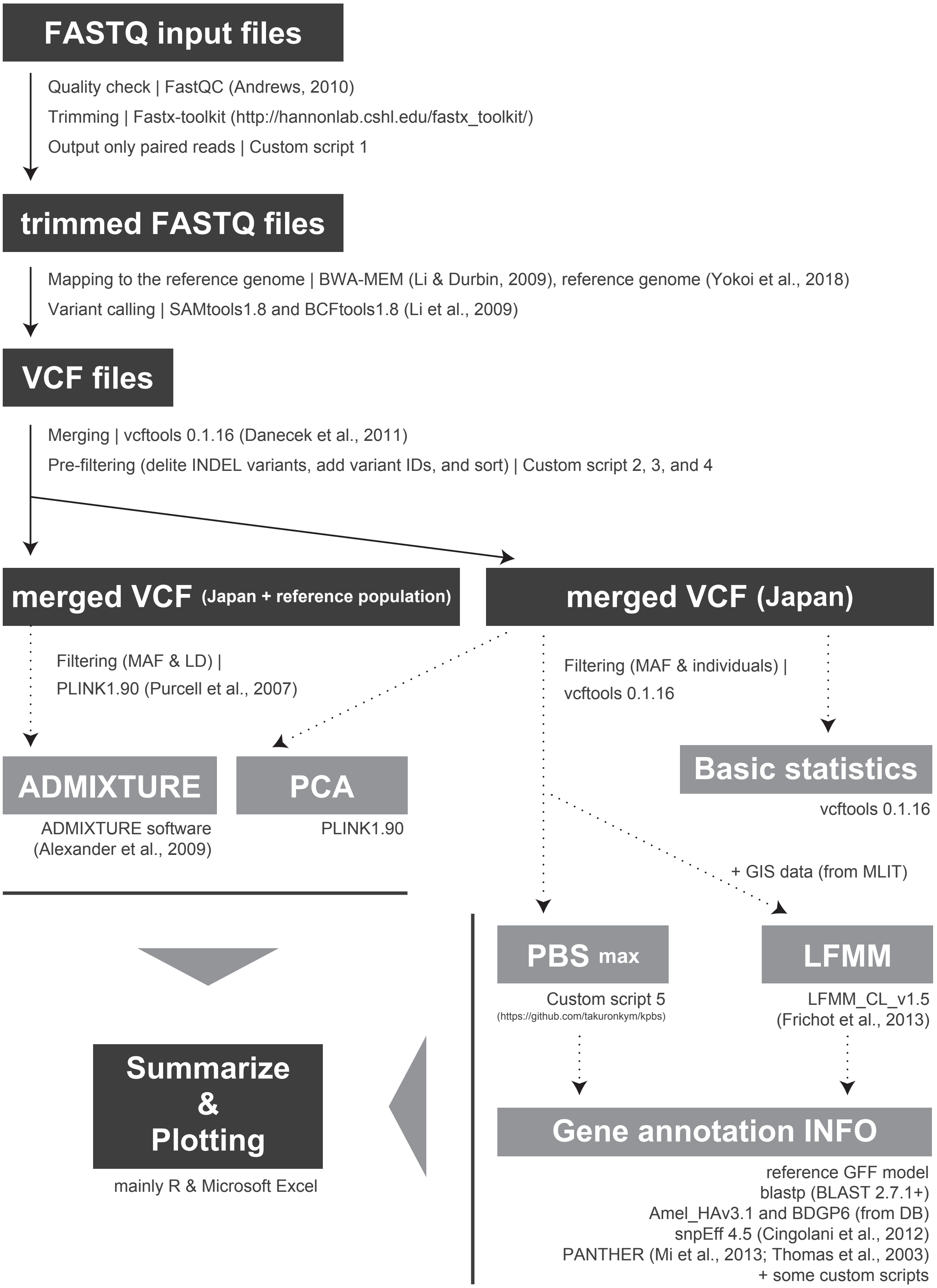

Supplement: Supplementary file 1 — Data S1: [file ECE3-13-e10573-s001.pdf]
